# Overexpression of BACH1 mediated by IGF2 facilitates hepatocellular carcinoma growth and metastasis via IGF1R and PTK2

**DOI:** 10.7150/thno.65775

**Published:** 2022-01-01

**Authors:** Meng Xie, Mengyu Sun, Xiaoyu Ji, Dongxiao Li, Xiaoping Chen, Bixiang Zhang, Wenjie Huang, Tongyue Zhang, Yijun Wang, Dean Tian, Limin Xia

**Affiliations:** 1 Department of Gastroenterology, Institute of Liver and Gastrointestinal Diseases, Hubei Key Laboratory of Hepato-Pancreato-Biliary Diseases, Tongji Hospital of Tongji Medical College, Huazhong University of Science and Technology, Wuhan 430030, Hubei Province, China; 2 Hubei Key Laboratory of Hepato-Pancreato-Biliary Diseases; Hepatic Surgery Center, Tongji Hospital, Tongji Medical College, Huazhong University of Science and Technology; Clinical Medicine Research Center for Hepatic Surgery of Hubei Province; Key Laboratory of Organ Transplantation, Ministry of Education and Ministry of Public Health, Wuhan, Hubei, 430030, China; 3 Department of Gastroenterology, Henan Provincial People's Hospital, Zhengzhou University People's Hospital, Zhengzhou 450004, Henan, China

**Keywords:** BTB and CNC homology 1, Insulin-like growth factor 1 receptor, Protein tyrosine kinase 2, Linsitinib, Defactinib

## Abstract

**Background:** Accumulating studies manifest that BTB and CNC homology 1 (BACH1) facilitates multiple malignancies progression and metastasis, and targeting the BACH1 pathway enhances antitumor efficacy. Nevertheless, the exact mechanism of BACH1 promoting growth and metastasis and its therapeutic significance in hepatocellular carcinoma (HCC) remain unclear.

**Methods:** The expression of BACH1 in human HCC specimens and HCC cell lines was analyzed by quantitative RT-PCR (RT-qPCR), western blot, and immunohistochemistry (IHC). The invasiveness and metastasis of HCC cells *in vitro* and *in vivo* were evaluated using transwell assays and orthotopic xenograft models. The luciferase reporter assays and chromatin immunoprecipitation (ChIP) assays were performed to explore the transcriptional regulation of insulin-like growth factor 1 receptor (*IGF1R*) and protein tyrosine kinase 2 (*PTK2*) by BACH1.

**Results:** BACH1 was prominently upregulated in human HCC samples and elevated BACH1 expression was associated with poor overall survival (OS) and high recurrence rates of HCC patients. BACH1 facilitated growth and metastasis of HCC by upregulating cell motility-related genes *IGF1R* and *PTK2*. Notably, insulin-like growth factor 2 (IGF2), the ligand of IGF1R, in turn upregulated BACH1 expression through the IGF1R-ERK1/2-ETS1 cascades, thus forming a positive feedback loop to provoke HCC growth and metastasis. Moreover, combining IGF1R inhibitor linsitinib with PTK2 inhibitor defactinib prominently suppressed BACH1-mediated HCC growth and metastasis.

**Conclusions:** These results demonstrated the tumorigenic and pro-metastatic role of BACH1 in HCC, which could be a promising biomarker for predicting poor prognosis and selecting patients who could benefit from combination therapy of IGF1R-targeted and PTK2-directed.

## Introduction

Hepatocellular carcinoma (HCC) remains a global health challenge, ranking as the sixth most common cancer and the fourth leading cause of cancer-related death 1. Patients with HCC have a poor prognosis, with a 5-year survival of 18% 2. Sorafenib has been the only available first-line treatment for advanced HCC for a decade 1. Whereafter, lenvatinib is approved as first-line therapy for advanced HCC 3. Nevertheless, both treatments have limited clinical benefits. Hence, new combination strategies are being developed. The combination of targeted therapies with immune checkpoint inhibitors, including atezolizumab with bevacizumab and lenvatinib with pembrolizumab, shows promising anti-tumor activity in unresectable HCC 4, 5. Based on the evidence of efficacy, atezolizumab plus bevacizumab has been offered as first-line therapy for HCC patients with advanced-stage 6. Numerous clinical trials exploring the efficacy of combination strategies are ongoing 1. Thus, a better understanding of the mechanisms rendering HCC malignant progression is pivotal to the development of effective combination therapies in HCC.

BTB and CNC homology 1 (BACH1), a basic leucine zipper transcription factor, regulates heme homeostasis, oxidative stress, cell cycle, hematopoiesis, and immunity 7. BACH1 is involved in the progression of multiple malignancies. In breast cancer, elevated BACH1 suggests poor prognosis and facilitates cell migration and invasion via regulating mitochondrial metabolism reprogramming and metastasis-associated genes, such as matrix metalloproteinase-9 (*MMP9*), vascular endothelial growth factor receptor (*VEGFR*), and C‐X‐C chemokine receptor type 4 (*CXCR4*) 8-10. Stabilization of BACH1 drives metastasis of lung cancer through activation of glycolysis-associated genes and pro-metastatic genes transcription 11, 12. BACH1 also acts as an oncogenic driver to promote colon cancer (CRC) progression and an independent factor in predicting CRC patients' survival and metastasis 13, 14. BACH1 promotes cancer cell metastasis in pancreatic cancer by controlling a set of epithelial-mesenchymal transition (EMT)-associated genes, including activating critical mesenchymal genes but repressing epithelial genes 15. In HCC, BACH1 functions as a cancer-promoting gene, facilitating cell proliferation, migration, and invasion 16. However, the exact mechanism of BACH1 promoting HCC malignant progression and its therapeutic significance in HCC remain unclear.

As a receptor tyrosine kinase, insulin-like growth factor 1 receptor (IGF1R) is activated by its ligand IGF2, regulating multiple physiological processes, including mammalian development, metabolism, and growth 17. Accumulating studies suggest that IGF2-IGF1R signaling is critical for cell proliferation, differentiation, EMT, migration, stemness maintenance, and drug resistance in multiple malignancies, including HCC 18, 19. IGF2-IGF1R signaling activation is enrichment in the proliferation subclass of HCC, which is more aggressive and has the worst prognosis 1, 20. Identifying IGF1R as a vital tumor-promoting protein stimulates the development of multiple IGF1R-targeted small-molecule inhibitors 18. However, the clinical efficacy of these inhibitors is limited. Thus, it is urgent to identify predictive biomarkers that could assist in selecting patients who could benefit from IGF1R-targeted therapies and exploit combination strategies to increase the efficacy of HCC therapy.

Protein tyrosine kinase 2 (PTK2) is both a non-receptor tyrosine kinase and an adaptor protein that governs fundamental processes in normal and cancer cells through its kinase-dependent or kinase-independent functions 21. PTK2 promotes malignancy by orchestrating a diverse range of cellular processes, such as cell survival, proliferation, migration, invasion, EMT, and angiogenesis 22. Overexpression of PTK2 is frequent and correlates with shorter overall survival and recurrence-free survival in HCC patients 23. PTK2 promotes cancer stem cells (CSCs) traits and drives tumorigenicity in HCC cells, which leads to HCC recurrence and sorafenib resistance 23. Knockdown of PTK2 in HCC cells reduces *in vitro* and *in vivo* tumorigenicity by inducing G2/M arrest and apoptosis, decreasing anchorage-independent growth, and modulating the expression of cancer-promoting genes 24. Combined PTK2 inhibitor and sorafenib reduce HCC growth by affecting tumor-promoting gene expression and inducing epigenetic changes 25. PTK2 may be a novel prognostic biomarker for HCC recurrence and a potential therapeutic target for human HCC treatment. Therefore, it is necessary to further study the mechanism of PTK2 overexpression in HCC and the therapeutic effect of PTK2 inhibitors in HCC.

Here, we report that BACH1 upregulates IGF1R and PTK2 to promote HCC growth and metastasis. In turn, IGF2/IGF1R signaling upregulates BACH1 expression through ERK1/2-ETS1 activation. This mechanistic study provides a rationale for the combination of IGF1R blockade and PTK2 inhibition as a more efficient therapy for HCC.

## Methods

### Orthotopic xenograft HCC model and *in vivo* treatment studies

BALB/C nude mice (Huafukang Biological Technology, Beijing, China) were housed on a 12 h light-dark cycle at 22 °C, and all experiments were approved by the ethics committee of Tongji Hospital of Tongji Medical College, Huazhong University of Science and Technology. For the orthotopic xenograft HCC model, the mixture of 4.0 × 10^6^ indicated HCC cells and Matrigel was orthotopically transplanted into the right liver lobe of nude mice (male, five weeks old). Every ten mice were randomly assigned to a group. Mice were injected with D-Luciferin (Gold Biotechnology, MO, USA) weekly for monitoring tumor growth and metastasis, and imaged with Lago X optical imaging system (SI Imaging, USA). At the endpoint, the mice were sacrificed and the lungs were stained with hematoxylin and eosin. For *in vivo* treatment studies, one week after transplantation of hepatoma cells, the mice were randomly divided into four groups and were respectively given phosphate-buffered saline (PBS), or linsitinib (40 mg/kg orally once daily), or defactinib (25 mg/kg orally twice daily), or the combination of both for eight weeks.

### The human Cell Motility RT^2^ profiler PCR array

The human Cell Motility RT^2^ profiler PCR array was used to detect the gene expression profiling of PLC/PRF/5-LV-BACH1 cells versus PLC/PRF/5-LV-Control cells and MHCC97H-shBACH1 cells versus MHCC97H-shControl cells. RNA extraction, DNase treatment, and RNA cleanup were conducted according to the manufacturer's protocol (Qiagen, Germany). The cDNA was synthesized by the RT^2^ First Strand Kit (Qiagen, Germany). Then the cDNA was mixed with 2 × RT^2^ qPCR SYBR Green Mastermix and ddH_2_O, and added to the PCR array 96-well plate (25 μL/well). The Bio-Rad CFX96 was used to perform a 2-step cycling program. The data was normalized by revising all Ct values according to the average Ct values of housekeeping genes present on the array. All the experiments were performed in triplicate.

### Reagents

PI3K inhibitor LY294002 (S1105), ERK inhibitor SCH772984 (S7101), IGF1R inhibitor linsitinib (OSI-906) (S1091), PTK2 inhibitor defactinib (VS-6063) (S7654), TAE226 (S2820) were purchased from Selleck Chemicals (Selleck, TX, USA). Recombinant human IGF2 (HY-P7019) was purchased from MedChemExpressAll (MCE, NY, USA). All reagents were used according to the manufacturer's instructions.

### Statistical analysis

Statistical analysis was conducted via SPSS software (Version 26.0) and GraphPad Prism 8.0 software. Differences between the data were analyzed by χ^2^ test and Student's t-tests. The survival curve was plotted by the Kaplan-Meier method (log-rank test). Univariate/multivariate analysis was determined by Cox proportional hazards regression models. Pearson correlation test was used to analyze correlations. P < 0.05 was considered statistically significant.

Additional material and method details are provided in the supplementary material.

## Results

### BACH1 is upregulated in human HCC samples and predicts poor prognosis

**Figure 1:**
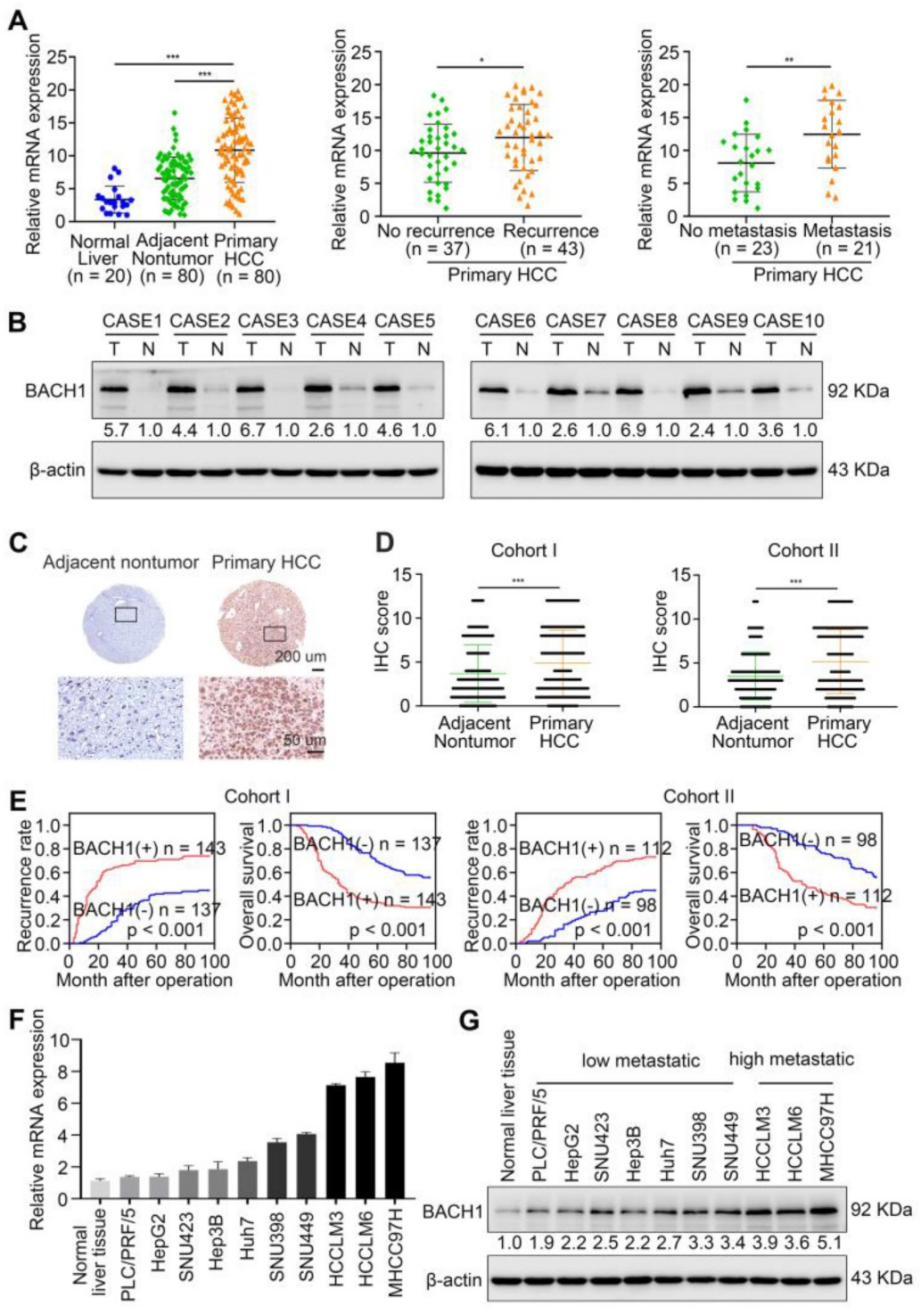

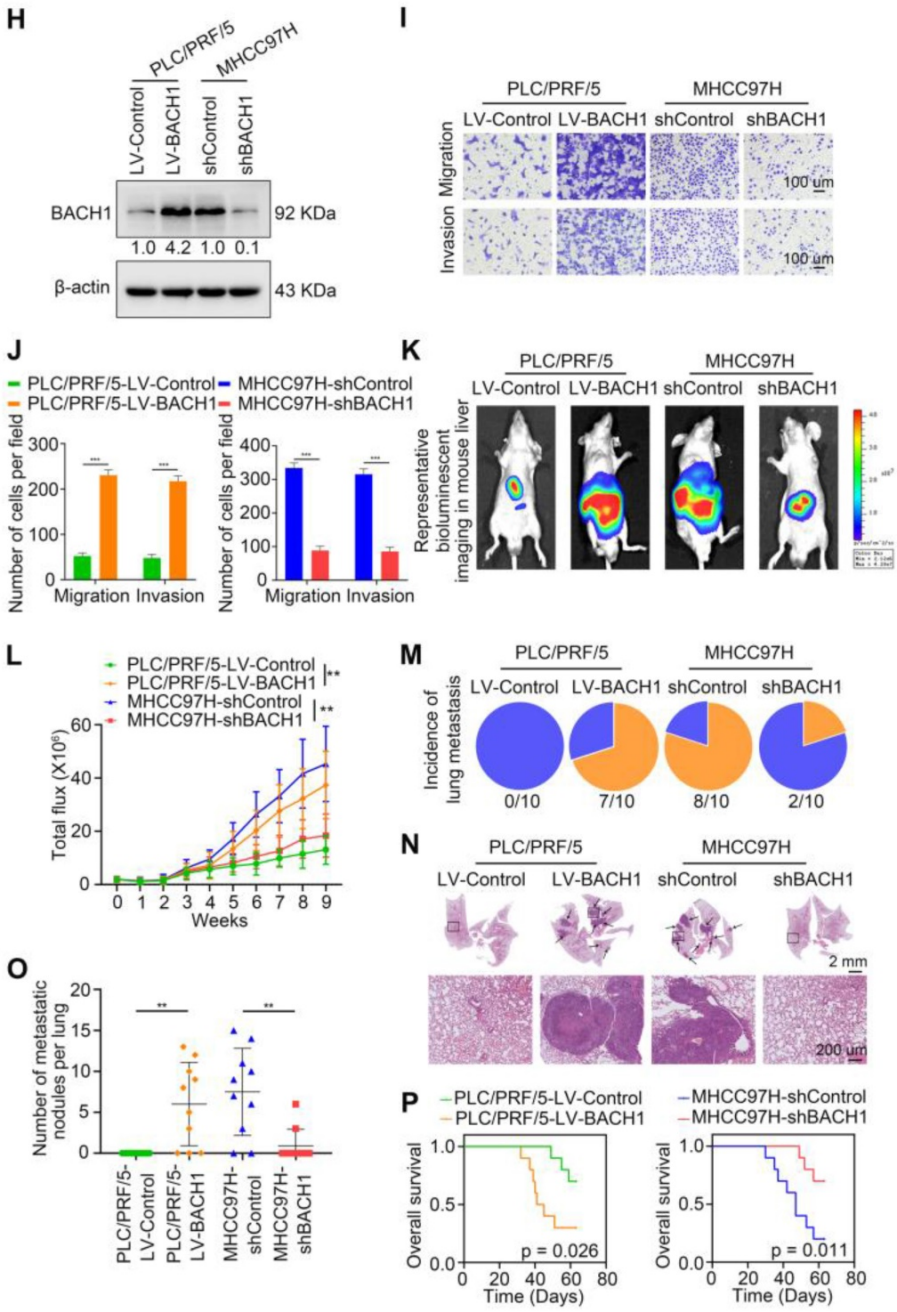
** BACH1 is upregulated and promotes tumor growth and metastasis in HCC. (A)** RT-qPCR analysis of *BACH1* mRNA levels in normal liver specimens (n = 20), adjacent nonneoplastic specimens (n = 80) and primary HCC specimens (n = 80) (left). *BACH1* mRNA levels in primary HCC patients without (n = 37) or with (n = 43) recurrence (middle). *BACH1* mRNA levels in primary HCC patients without (n = 23) or with (n = 21) metastasis (right). **(B)** Western blot analysis of BACH1 protein levels in primary HCC specimens and adjacent nonneoplastic specimens (n = 10). **(C-D)** Representative IHC staining (C) and IHC scores (D) of BACH1 in matched nonneoplastic specimens and primary HCC specimens from two different HCC cohorts (Cohort I = 280; Cohort II = 210). Scale bars, 200 µm (upper), 50 µm (lower). **(E)** The correlation between BACH1 expression and OS or recurrence rates of HCC patients was analyzed by Kaplan-Meier. **(F-G)** The mRNA (F) and protein (G) levels of BACH1 in normal liver specimens and HCC cells with different metastatic abilities. **(H)** Western blot verifying BACH1 overexpression (LV-BACH1) and knockdown (shBACH1) effect in PLC/PRF/5 and MHCC97H cells. **(I-J)** The migratory and invasive capacity of PLC/PRF/5-LV-BACH1, MHCC97H-shBACH1 and corresponding control cells were detected by transwell assay. Scale bar, 100 µm. **(K-P)** The effects of BACH1 on growth and metastasis in HCC orthotopic xenograft models. The representative bioluminescent imaging in the liver (K), the bioluminescent signals of liver tumors (L), the occurrence of pulmonary metastasis (M), the representative H&E staining images of pulmonary tissues (N), the number of pulmonary metastasis lesions (O) from nude mice after orthotopic transplantation with PLC/PRF/5-LV-BACH1, MHCC97H-shBACH1 and corresponding control cells were shown. The OS of mice was analyzed in (P). Scale bars, 2 mm (upper), 200 µm (lower). *p < 0.05, **p < 0.01, ***p < 0.001. Data were shown as Mean ± SD.

First, the mRNA levels of *BACH1* were detected in 20 normal liver specimens and 80 paired fresh primary HCC specimens by quantitative RT-PCR (RT-qPCR). Compared with normal liver and adjacent nonneoplastic tissues, *BACH1* mRNA levels were increased in primary HCC tissues (Figure 1A left). Furthermore, the levels of *BACH1* were elevated in relapsed patients compared to non-relapsed patients (Figure 1A middle). Notably, *BACH1* was prominently overexpressed in metastatic patients than non-metastatic patients (Figure 1A right).

Then, we evaluated the protein levels of BACH1 in paired fresh HCC samples. Western-blot assay showed that BACH1 was prominently elevated in HCC specimens than adjacent nonneoplastic specimens (Figure 1B). Consistently, immunohistochemical (IHC) staining suggested that BAHC1 was obviously upregulated in tumor tissues compared to adjacent nonneoplastic tissues in two HCC cohorts (Figure 1C-D, Table S9, S10).

To investigate the clinical significance of BACH1, HCC patients were categorized into two groups based on IHC examination: the negative group (patients with negative BACH1 expression) and the positive group (patients with positive BACH1 expression). Notably, Kaplan-Meier analysis revealed that higher BACH1 levels correlated with worse OS and higher recurrence rates in HCC patients (Figure 1E). The elevated levels of BACH1 were related to increased tumor number, larger tumor size, tumor encapsulation loss, microvascular invasion, less differentiation, and poor tumor-nodule-metastasis (TNM) staging (Table 1). Multivariate analysis indicated that BACH1 was a significant and independent predictor of relapse and survival of HCC patients (Table S1, S2). Altogether, our data indicated that BACH1 was upregulated and predicted poor prognosis in HCC patients.

### BACH1 accelerates HCC growth and metastasis

To further investigate the roles of BACH1 in regulating the malignant process of HCC, we detected the levels of BACH1 in different HCC cells. Our results indicated that compared with HCC cells with low metastatic ability, the levels of BACH1 were enhanced in HCC cells with high metastatic ability (Figure 1F-G). Accordingly, we overexpressed BACH1 in PLC/PRF/5 cells with low BACH1 levels (Figure 1H). To silence the BACH1 expression, we generated three BACH1-specific short hairpin RNAs (Figure S1A). Based on the knockdown effect, the shBACH1#2 was adopted for knocking down the BACH1 expression in MHCC97H cells that highly express BACH1 in the following studies (Figure S1A, Figure 1H). The results suggested that BACH1 overexpression augmented the proliferation of PLC/PRF/5 cells, and BACH1 silencing attenuated the proliferation of MHCC97H cells *in vitro* (Figure S1 D-E). Moreover, overexpression of BACH1 led to a dramatic increase of the migratory and invasive capacity of PLC/PRF/5 cells, while knockdown of BACH1 strikingly mitigated the migration and invasion of MHCC97H cells *in vitro* (Figure 1I-J, Figure S1 B-C). To further verify these observations, we overexpressed BACH1 in Hep3B cells that lowly express BACH1 and knocked down BACH1 expression in HCCLM3 cells that highly express BACH1 (Figure 1F-G, Figure S2A, S2D). The transwell assay showed that overexpression of BACH1 significantly enhanced the migration and invasion abilities of Hep3B cells, while knockdown of BACH1 led to a dramatic inhibition of migration and invasion of HCCLM3 cells (Figure S2B-C, S2E-F). These results further confirmed that BACH1 promoted HCC metastasis.

To further validate this observation, we established orthotopic growth and metastatic models to examine the effects of BACH1 on HCC progression *in vivo*. In subcutaneous xenograft models, overexpression of BACH1 increased the volume and weight of tumors, while BACH1 silencing decreased the growth of tumors (Figure S1 F-G). In orthotopic xenograft models, we found that BACH1 overexpression augmented tumor growth and BACH1 silencing alleviated tumor growth in the liver (Figure 1K-L). The occurrence of pulmonary metastasis and the number of pulmonary metastasis lesions were much higher, and the OS was much shorter in the BACH1 overexpression group than in the control group (Figure 1M-P). In contrast, knockdown of BACH1 decreased tumor growth and lung metastasis but increased OS (Figure 1M-P). Collectively, our findings demonstrated that BACH1 accelerated HCC growth and metastasis.

### BACH1 regulates cancer progression-associated genes, especially *IGF1R* and *PTK2*

**Figure 2:**
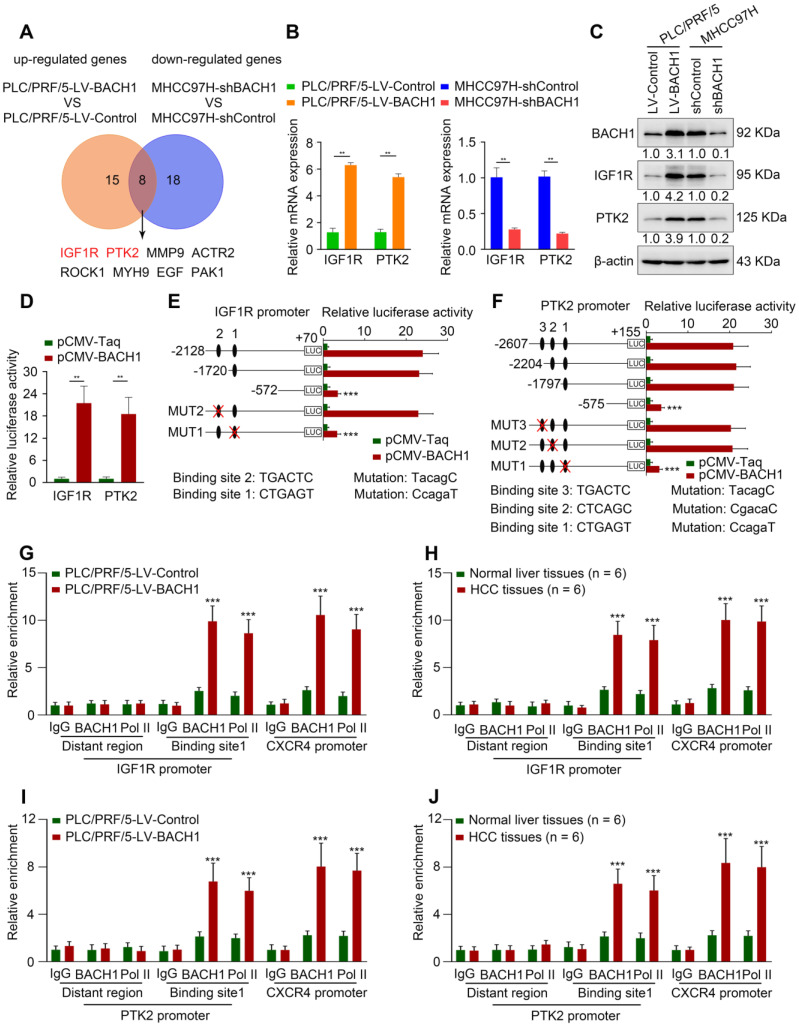
** BACH1 regulates cell motility-associated genes *IGF1R* and *PTK2* expression. (A)** The overlap of up-regulated genes in PLC/PRF/5-LV-BACH1 cells VS PLC/PRF/5-LV-Control cells and down-regulated genes in MHCC97H-shBACH1 cells VS MHCC97H-shControl cells (fold change ≥ 2.0) were detected by the Human Cell Motility PCR Array and showed by Venn diagrams. **(B-C)** The effects of BACH1 overexpression or knockdown on IGF1R and PTK2 expression. The mRNA (B) and protein (C) levels of IGF1R and PTK2 in the PLC/PRF/5-LV-BACH1, MHCC97H-shBACH1 and corresponding control cells were determined by RT-qPCR and western blot. **(D)** Luciferase activities of *IGF1R* and *PTK2* promoter reporter vectors in the PLC/PRF/5 cells cotransfected with pCMV-BACH1 or pCMV-Taq. **(E-F)** Luciferase activities of serially truncated/mutated *IGF1R* (E) and *PTK2* (F) promoters reporter vectors in the PLC/PRF/5 cells cotransfected with pCMV-BACH1or pCMV-Taq. **(G-J)** ChIP analysis of BACH1 or RNA polymerase II (Pol II) occupancy in the *IGF1R*, *PTK2* or *CXCR4* promoter region in the indicated PLC/PRF/5 cells and HCC tissues (n = 6). An isotype-matched IgG was used as a negative control. *CXCR4* promoter region and Pol II were used as positive controls. *P < 0.05, **P < 0.01, ***P < 0.001. Data were shown as Mean ± SD.

Cell motility is a critical step in the progression of malignancy. To elucidate the precise mechanism underlying the tumorigenic and pro-metastatic role of BACH1 in HCC, we used a Human Cell Motility PCR Array to detect the mRNA expression profile after BACH1 expression change. Among 84 cell motility-related genes, 15 genes were up-regulated in PLC/PRF/5-LV-BACH1 cells versus control cells, while 18 genes were down-regulated in MHCC97H-shBACH1 cells than control cells (fold change ≥ 2) (Table S3, S4). The overlapping genes included *IGF1R*, *PTK2*, *MMP9*, *ACTR2*, *ROCK1*, *MYH9*, *EGF*, and *PAK1*. *IGF1R* and *PTK2* caught our attention because they were the most strongly changed (Figure 2A, Table S3, S4). TCGA-LIHC database indicated that *IGF1R* and *PTK2* expression positively correlated with *BACH1* expression (Figure S3; http://gepia.cancer-pku.cn/; http://timer.cistrome.org/). Upregulation of IGF1R and PTK2 after BACH1 overexpression was validated in PLC/PRF/5 cells (Figure 2B-C). Conversely, knockdown of BACH1 markedly attenuated the levels of IGF1R and PTK2 in MHCC97H cells (Figure 2B-C).

To determine whether *IGF1R* and *PTK2* are direct BACH1 targets, the luciferase reporter assay was performed, and we found that overexpression of BACH1 increased *IGF1R* and *PTK2* promoter activities (Figure 2D). Next, we analyzed potential BACH1 binding sites within the promoter regions of *IGF1R* and *PTK2*. The results showed that there were two and three putative BACH1 binding motifs within *IGF1R* and *PTK2* promoters, respectively (Figure S4, S5). Subsequently, we constructed a series of truncated or mutant *IGF1R* and *PTK2* promoter plasmids. The *IGF1R* luciferase reporter activity induced by BACH1 was noticeably decreased under deletion of the region between -1720 bp to -572 bp, suggesting this region was vital for BACH1-induced IGF1R expression (Figure 2E). Congruously, the binding site 1 mutation in this region abolished the activity of the *IGF1R* luciferase reporter (Figure 2E). Similarly, deletion of sequence between -1797 bp to -575 bp significantly disrupted the *PTK2* luciferase reporter activity induced by BACH1 (Figure 2F). The *PTK2* luciferase reporter activity was declined upon mutation of binding site 1 in this sequence (Figure 2F). Finally, the chromatin immunoprecipitation (ChIP) analysis suggested that BACH1 occupied the promoter regions of *IGF1R* and *PTK2* in PLC/PRF/5-LV-BACH1 cells and human HCC specimens (Figure 2G-J). These data manifested that BACH1 directly activated *IGF1R* and *PTK2* transcription.

### Direct upregulation of IGF1R and PTK2 facilitates the tumorigenic and pro- metastatic properties of BACH1

As a receptor tyrosine kinase, IGF1R promotes proliferation, migration, and metastasis in numerous malignant tumors 18. PTK2 facilitates tumor progression and metastasis via its kinase activity and scaffolding function 22. Thus, we investigated whether BACH1 promotes HCC growth and metastasis by directly upregulating IGF1R and PTK2.

**Figure 3:**
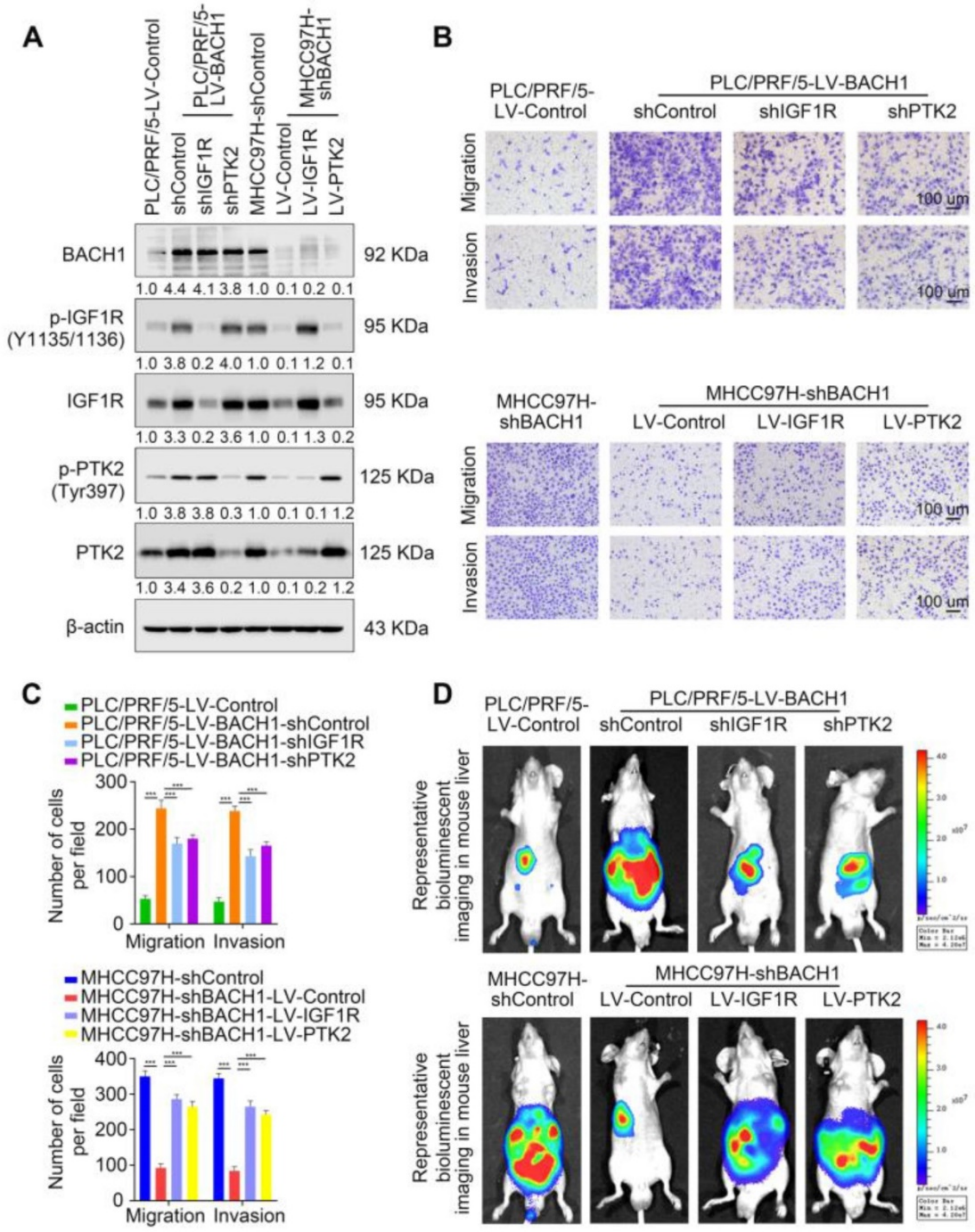

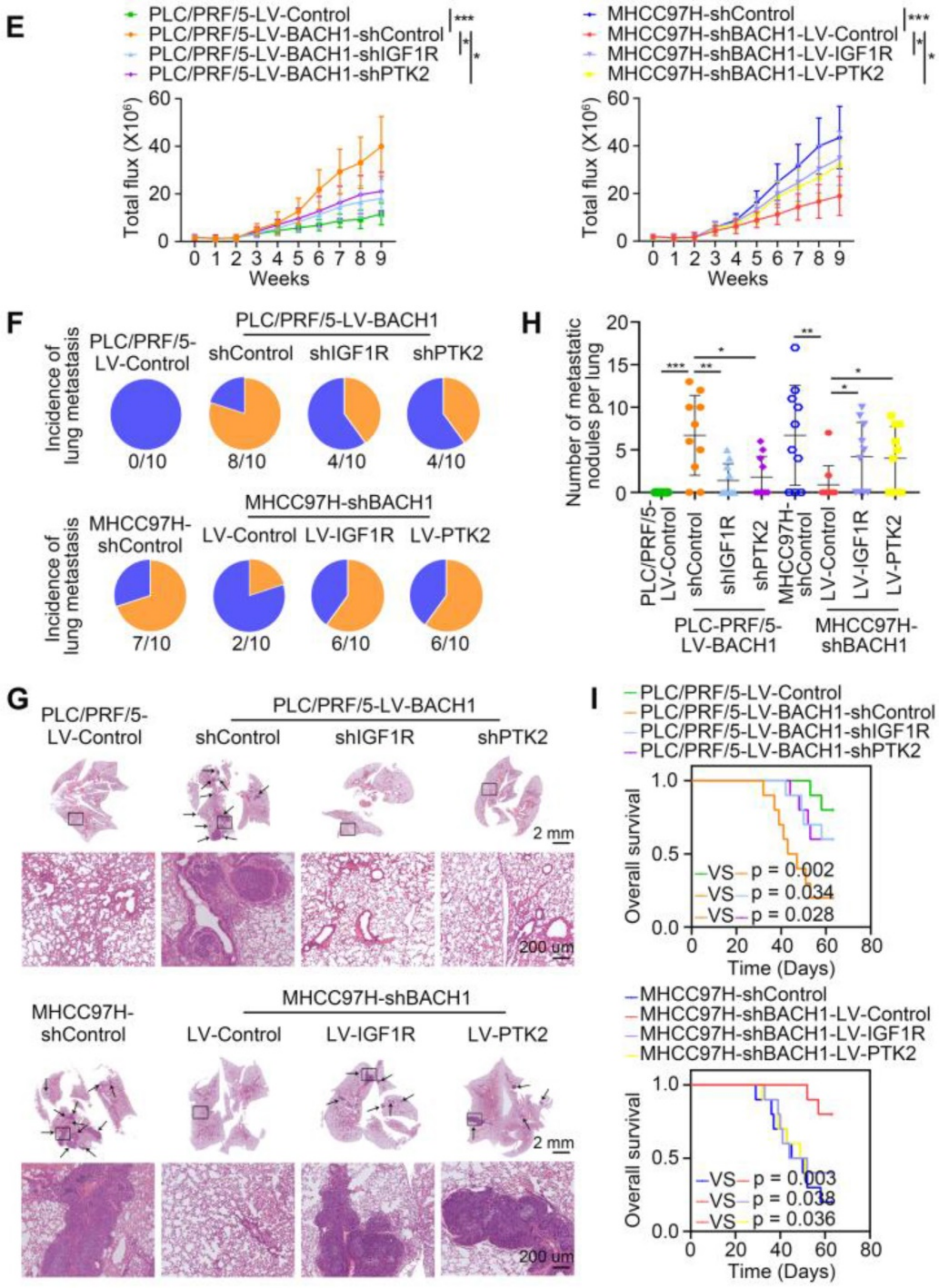
** BACH1 accelerates HCC growth and metastasis via directly upregulating IGF1R and PTK2. (A)** IGF1R and PTK2 were knocked down in BACH1-overexpressing PLC/PRF/5 cells or overexpressed in BAHC1-knockdown MHCC-97H cells. The protein levels of BACH1, p-IGF1R, IGF1R, p-PTK2 and PTK2 in the indicated HCC cells were determined by western blot. **(B-C)** The migratory and invasive capacity of the indicated PLC/PRF/5 and MHCC97H cells were detected by transwell assay. Scale bar, 100 µm. **(D-I)** HCC orthotopic xenograft models indicated that BACH1 promotes HCC growth and metastasis by upregulating IGF1R and PTK2. The representative bioluminescent imaging in the liver (D), the bioluminescent signals of liver tumors (E), the occurrence of pulmonary metastasis (F), the representative H&E staining images of pulmonary tissues (G), the number of pulmonary metastasis lesions (H) from nude mice after orthotopic transplantation with indicated PLC/PRF/5 and MHCC97H cells were shown. The OS of mice was analyzed in (I). Scale bars, 2 mm (upper), 200 µm (lower). *p < 0.05, **p < 0.01, ***p < 0.001. Data were shown as Mean ± SD.

First, we knocked down the expression of IGF1R or PTK2 in PLC/PRF/5-LV-BACH1 cells (Figure S6A-B, Figure 3A). Moreover, we overexpressed IGF1R or PTK2 in the MHCC97H-shBACH1 cells (Figure 3A). Transwell assays indicated that IGF1R and PTK2 silencing significantly attenuated the increased migratory and invasive capacity of PLC/PRF/5-LV-BAHC1 cells, while upregulating IGF1R and PTK2 rescued the migratory and invasive capacity of MHCC97H-shBACH1 cells (Figure 3B-C). Consistently, silencing IGF1R or PTK2 decreased tumor growth in the liver, the occurrence of pulmonary metastasis, and the number of pulmonary metastasis lesions, resulting in prolonged OS of nude mice bearing orthotopic xenografts of PLC/PRF/5-LV-BACH1 cells (Figure 3D-I). Conversely, the growth of tumors in the liver, the occurrence of pulmonary metastasis, and the number of pulmonary metastasis lesions were much higher in the IGF1R or PTK2 overexpression group than those in the control group, which led to shortened OS (Figure 3D-I). Taken together, these results suggested that IGF1R and PTK2 mediated the tumorigenic and pro-metastatic properties of BACH1.

### IGF2/IGF1R signaling regulates BACH1 expression through ERK1/2 activation

Considering the importance of BACH1 in tumorigenesis and promoting metastasis, we next probed the molecular mechanism of BACH1 upregulation in HCC. Because IGF1R contributed to the aggressive properties of BACH1, one of its ligands, IGF2, attracted our attention. Unlike IGF1, which is decreased in HCC and whose lower levels may be a potential risk factor for HCC progression, IGF2 is significantly increased in HCC and serum IGF2 levels are associated with extrahepatic metastasis 26, 27. IGF2 induces HCC cell migration, invasion, and lung metastases 19. Of note, IGF2 regulates complex gene expression profiles in HCC cells, including cell adhesion and motility-related genes, hepatocarcinogenesis-associated genes, IGF signaling-related genes, etc 28. Given that both IGF2 and BACH1 play vital roles in HCC progression and IGF2 induces gene regulation in HCC cells, it is tempting to explore whether IGF2 regulates BACH1 expression.

**Figure 4:**
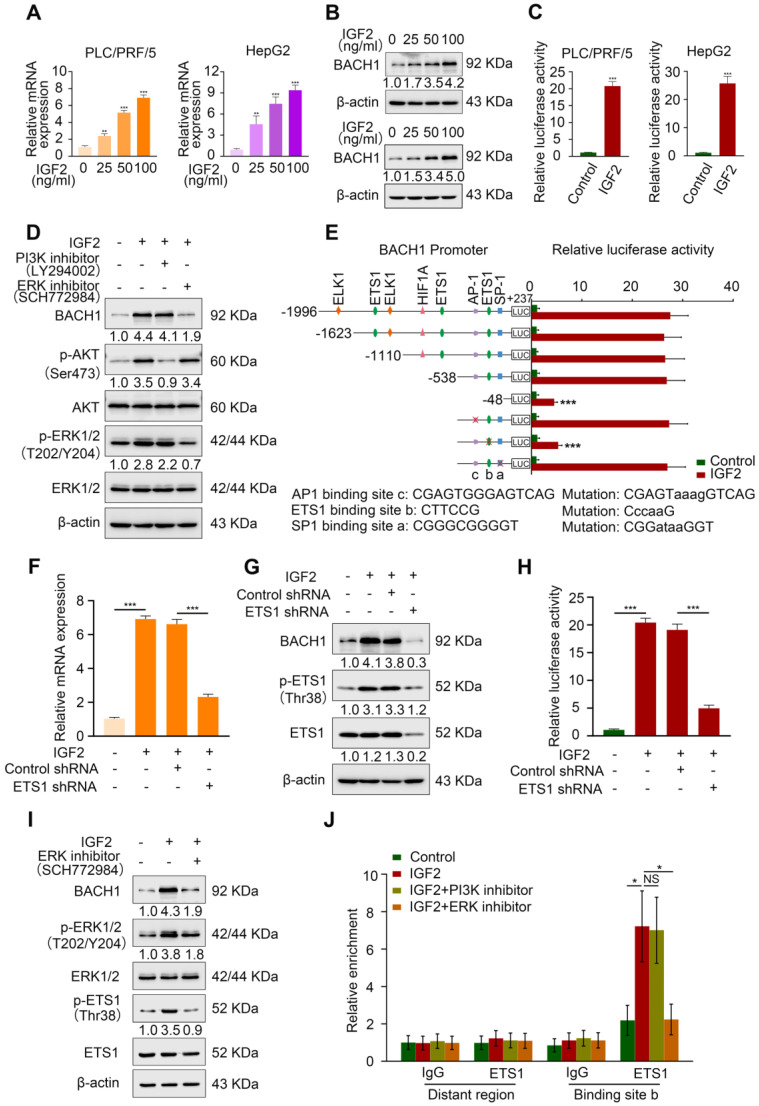
** IGF2 facilitates BACH1 upregulation via the ERK1/2/ETS1 pathway. (A-B)** The mRNA and protein level of BACH1 when PLC/PRF/5 (A-left, B-upper) and HepG2 cells (A-right, B-lower) were treated with IGF2 at the 0 ng/ml, 25 ng/ml, 50 ng/ml, and 100 ng/ml for 24 h. **(C)** The relative luciferase activities of *BACH1* promoter reporter vectors in PLC/PRF/5 cells (left) and HepG2 cells (right) treated with IGF2 (100 ng/ml) for 24 h were analyzed by luciferase reporter assays. **(D)** The protein levels of BACH1, p-AKT, AKT, p-ERK1/2, and ERK1/2 in PLC/PRF/5 cells treated with PI3K inhibitor (LY294002, 20 μM) or ERK inhibitor (SCH772984, 100 nM) in the presence of IGF2 (100 ng/ml, 24 h). **(E)** The serially truncated/mutated BACH1 luciferase reporter activities in the PLC/PRF/5 cells treated with or without IGF2 (100 ng/ml, 24 h). **(F-H)** The PLC/PRF/5 cells were transfected with shETS1 or control shRNA in the presence of IGF2 (100 ng/ml, 24 h). The mRNA expression of BACH1 was detected by RT-qPCR (F). The protein levels of BACH1, ETS1 and p-ETS1 were detected by western blot (G). The relative luciferase activities of *BACH1* promoter reporter vectors were analyzed by luciferase reporter assays (H). **(I)** The effects of ERK inhibitor on BACH1, p-ERK1/2, ERK1/2, p-ETS1, and ETS1 levels in PLC/PRF/5 cells treated with IGF2 (100 ng/ml, 24 h). **(J)** ChIP analysis of ETS1 binding to the *BACH1* promoter region in PLC/PRF/5 cells treated with PI3K inhibitor or ERK inhibitor in the presence of IGF2 (100 ng/ml, 24 h). *p < 0.05, **p < 0.01, ***p < 0.001, NS: no statistical difference. Data were shown as Mean ± SD.

First, we treated PLC/PRF/5 cells and HepG2 cells, which also have a low expression of BACH1, with different concentrations of recombinant IGF2 for 24 h. IGF2 upregulated BACH1 in a dose-dependent manner (Figure 4A-B). Luciferase reporter assay showed that IGF2 treatment markedly increased BACH1 promoter activity (Figure 4C). IGF2/IGF1R signaling activates two main signaling pathways: PI3K/AKT and Ras/Raf/ERK1/2 cascades 29. Next, to define which signaling pathway is involved in IGF2-induced BACH1 upregulation, PLC/PRF/5 cells were treated with PI3K inhibitor LY294002 and ERK1/2 inhibitor SCH772984. Treatment of ERK1/2 inhibitor significantly abolished IGF2-induced BACH1 expression, whereas PI3K inhibitor had no such effect (Figure 4D). These results indicated that ERK1/2 signaling contributed to IGF2-induced BACH1 expression.

To further clarify the mechanism of IGF2-induced BACH1 expression, we analyzed the *BACH1* promoter sequence and identified several potential binding sites of transcription factors involved in the ERK1/2 signaling (Figure S7). Subsequently, we designed a series of truncation/mutations of the *BACH1* promoter sequence. Deletion of the region from -538 bp to -48 bp markedly decreased the *BACH1* luciferase reporter activity induced by IGF2, suggesting this region was vital for IGF2-induced BACH1 expression (Figure 4E). Congruously, mutation of a putative ETS1 binding site in this region attenuated the *BACH1* promoter activity, while mutations of AP-1 and SP-1 binding sites in this region had no such effect (Figure 4E). To further validate this result, we knocked down ETS1 expression in PLC/PRF/5 cells (Figure S6C). Knockdown of ETS1 significantly abolished the upregulated *BACH1* expression and the increased activity of *BACH1* promoter induced by IGF2 (Figure 4F-H). Moreover, ERK inhibitor (SCH772984) simultaneously decreased the levels of BACH1, p-ERK1/2, and p-ETS1 in IGF2-treated PLC/PRF/5 cells, indicating that the ERK1/2-ETS1 signaling was indispensable for IGF2-induced BACH1 expression (Figure 4I). Finally, the ChIP assay showed that ERK1/2 inhibitor reduced the relative enrichment of ETS1 on the *BACH1* promoter in IGF2-treated PLC/PRF/5 cells, while PI3K inhibitor had no such effect (Figure 4J). Altogether, these results demonstrated that IGF2/IGF1R signaling transactivated *BACH1* via IGF2/IGF1R-ERK1/2-ETS1 cascades.

### Oncogenic IGF2 depends on BACH1 to promote HCC growth and metastasis

**Figure 5:**
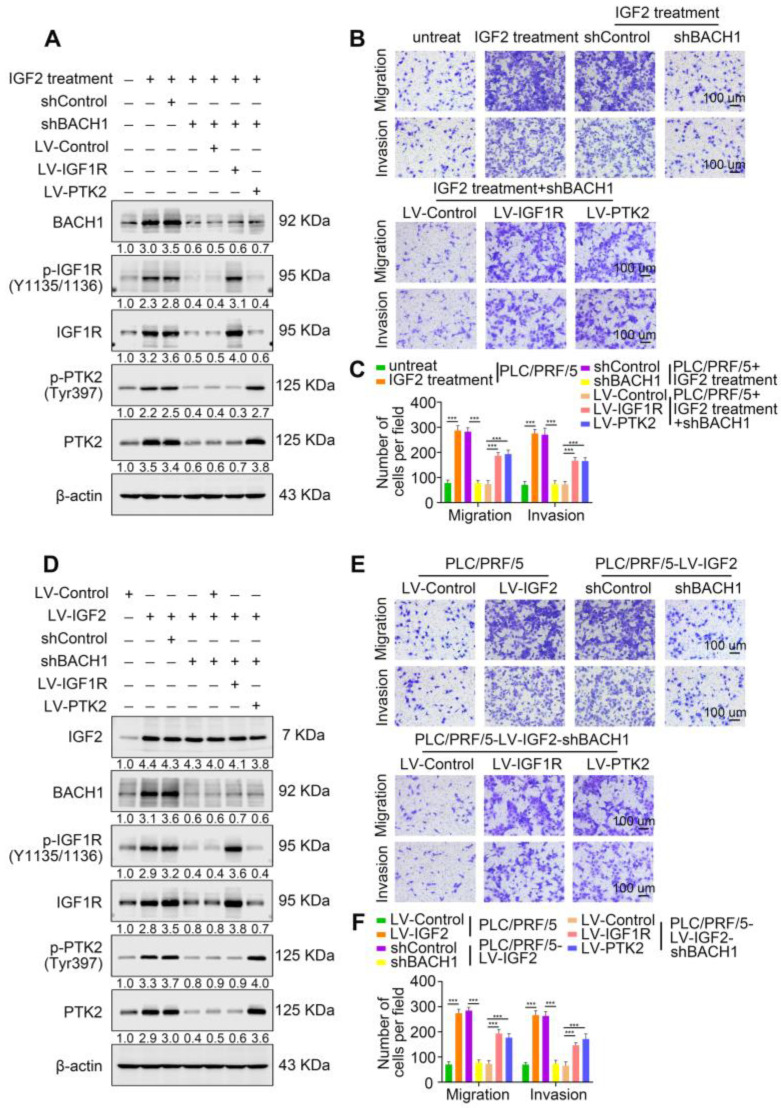

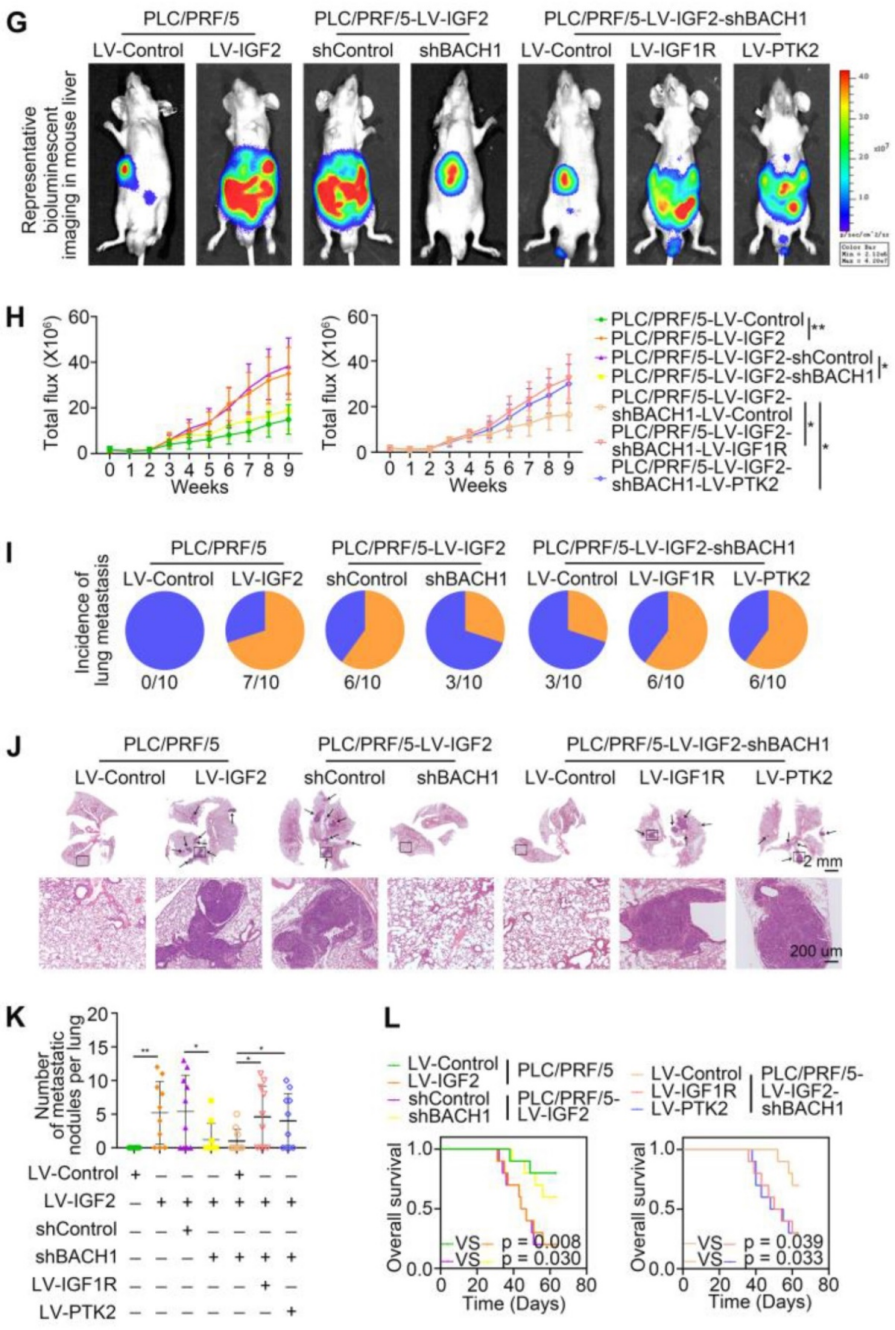
** IGF2 promotes HCC growth and metastasis depending on BACH1. (A)** BACH1 was silenced in PLC/PRF/5 cells. IGF1R and PTK2 were overexpressed in BACH1-silencing PLC/PRF/5 cells. These cells were treated with IGF2 at 100 ng/ml for 24 h. The protein levels of BACH1, p-IGF1R, IGF1R, p-PTK2 and PTK2 in the indicated PLC/PRF/5 cells were detected by western blot. **(B-C)** The migratory and invasive capacity of the indicated PLC/PRF/5 cells were analyzed by transwell assay. Scale bar, 100 µm. **(D)** IGF2 was overexpressed in PLC/PRF/5 cells and then BACH1 was silenced in IGF2-overexpressing PLC/PRF/5 cells. IGF1R and PTK2 were overexpressed in BACH1-silencing PLC/PRF/5-LV-IGF2 cells. The protein levels of IGF2, BACH1, p-IGF1R, IGF1R, p-PTK2 and PTK2 in the indicated PLC/PRF/5 cells were detected by western blot. **(E-F)** The migratory and invasive capacity of the indicated PLC/PRF/5 cells were detected by transwell assay. Scale bar, 100 µm. **(G-L)** The indicated HCC cells were used to construct HCC orthotopic xenograft models. The representative bioluminescent imaging in the liver (G), the bioluminescent signals of liver tumors (H), the occurrence of pulmonary metastasis (I), the representative H&E staining images of pulmonary tissues (J), the number of pulmonary metastasis lesions (K) from nude mice after orthotopic transplantation with indicated PLC/PRF/5 cells were shown. The OS of mice was analyzed in (L). Scale bars, 2 mm (upper), 200 µm (lower). *p < 0.05, **p < 0.01, ***p < 0.001. Data were shown as Mean ± SD.

Accumulating studies manifest that IGF2 facilitates HCC growth and metastasis 28, 30. Since BACH1 was upregulated by IGF2 and accelerated HCC growth and metastasis, we considered whether BACH1 contributed to IGF2-induced HCC progresssion. We silenced BACH1 in PLC/PRF/5 cells and then treated PLC/PRF/5 and PLC/PRF/5-shBACH1 cells with recombinant IGF2 (Figure 5A). The western blot assay showed that IGF2 treatment increased the expression of BACH1, IGF1R and PTK2 in PLC/PRF/5 cells, while BACH1 silencing attenuated the increased expression of BACH1, IGF1R and PTK2 induced by IGF2 (Figure 5A). These results suggested that IGF2 affected the expression of BACH1, IGF1R and PTK2, and that its effect on IGF1R and PTK2 was mediated by BACH1. In addition, IGF2 treatment led to a dramatic increase of migratory and invasive capacity of PLC/PRF/5 cells, whereas silencing BACH1 diminished the increased cell migration and invasion induced by IGF2 (Figure 5B-C). Considering that IGF1R and PTK2 mediated the tumorigenic and pro-metastatic properties of BACH1, it is necessary to investigate whether overexpression of IGF1R and PTK2 could rescue the inhibition of migration and invasion caused by BACH1-depletion. Thus, we overexpressed IGF1R and PTK2 in PLC/PRF/5-shBACH1 cells and then treated them with recombinant IGF2 (Figure 5A). Transwell assays suggested that overexpression of IGF1R and PTK2 rescued the attenuated cell migration and invasion induced by BACH1-silencing (Figure 5B-C). To further confirm the above observations, we overexpressed IGF2 in PLC/PRF/5 cells and then silenced BACH1 in PLC/PRF/5-LV-IGF2 cells (Figure 5D). In addition, we overexpressed IGF1R and PTK2 in PLC/PRF/5-LV-IGF2-shBACH1 cells (Figure 5D). Transwell assays indicated that overexpression of IGF2 significantly augmented the migratory and invasive capacity of PLC/PRF/5 cells, silencing BACH1 attenuated the increased cell migration and invasion induced by IGF2, while overexpression of IGF1R and PTK2 rescued the inhibition of migration and invasion induced by BACH1- depletion (Figure 5E-F). Consistent with these results *in vitro*, the growth of tumors in the liver, the occurrence of pulmonary metastasis, and the number of pulmonary metastasis lesions were much higher in the IGF2 overexpression group than those in the control group, resulting in shorter OS of nude mice bearing orthotopic xenografts of PLC/PRF/5-LV-IGF2 cells (Figure 5G-L). Nevertheless, knockdown of BACH1 dramatically abolished the increased tumor growth in the liver, the occurrence of pulmonary metastasis, and the number of pulmonary metastasis lesions, leading to prolonged OS (Figure 5G-L). Overexpression of IGF1R and PTK2 rescued the inhibition of tumor growth and metastasis caused by BACH1-depletion (Figure 5G-L). Collectively, these results suggested that IGF2 promoted HCC progression depending on BACH1-induced IGF1R and PTK2 expression.

### Overexpression of BACH1 correlates with increased levels of IGF1R and PTK2 in HCC tissues

**Figure 6:**
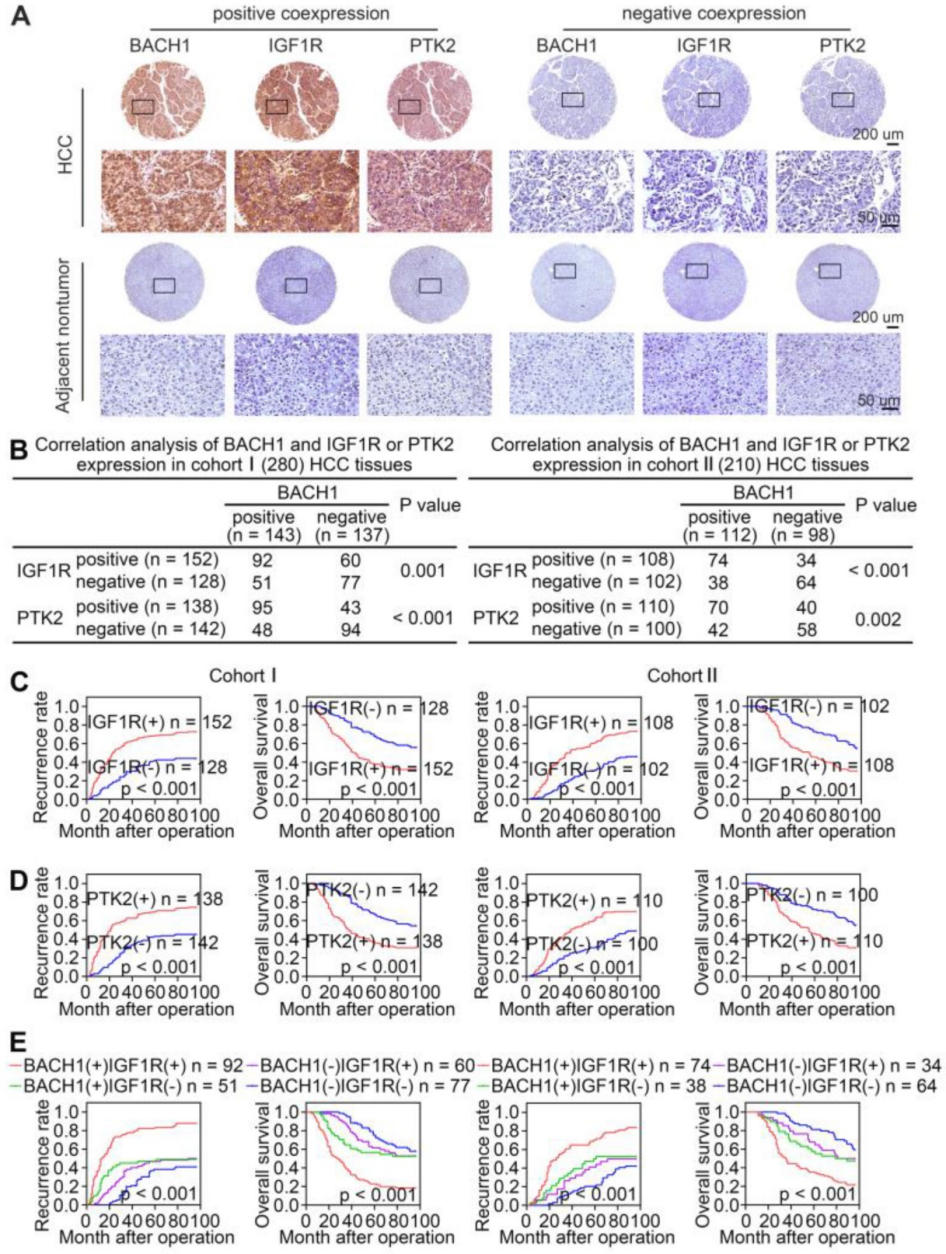

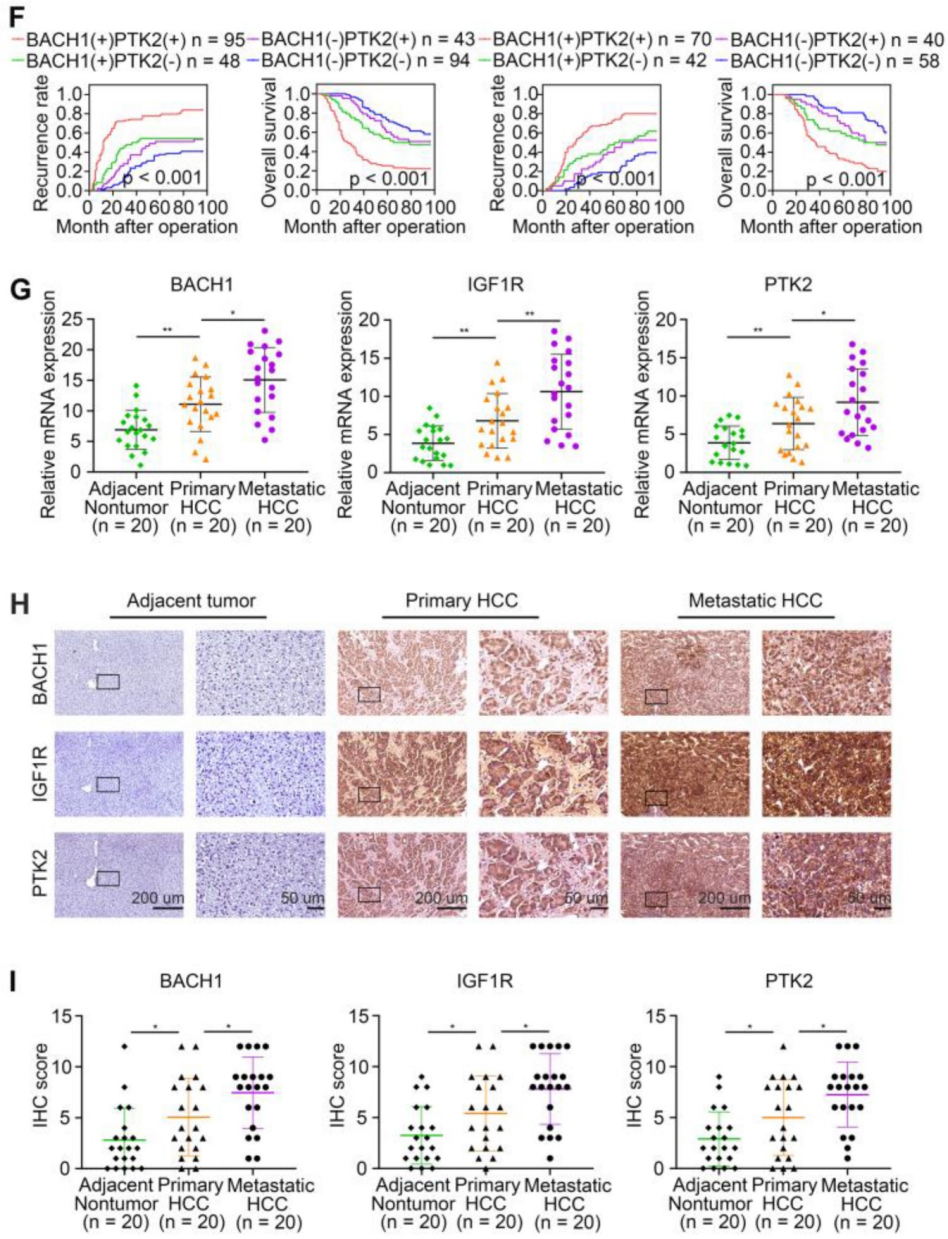
** The levels of BACH1 positively correlates with IGF1R and PTK2 levels in human HCC tissues. (A)** Representative IHC staining of BACH1, IGF1R, and PTK2 in primary HCC tissues and matched nonneoplastic tissues were shown. Scale bars, 200 µm (upper), 50 µm (lower). **(B)** The correlation analysis of BACH1 and IGF1R or PTK2 levels in two HCC cohorts. **(C-F)** The correlations between IGF1R, PTK2, BACH1/IGF1R, or BACH1/PTK2 expression and OS or recurrence rates were analyzed by Kaplan-Meier. **(G)** Relative mRNA expression of *BACH1*, *IGF1R* and *PTK2* in adjacent nonneoplastic specimens (n = 20), primary HCC specimens (n = 20) and metastatic HCC specimens (n = 20) were analyzed by RT-qPCR. **(H-I)** Representative IHC staining (H) and IHC scores (I) of BACH1, IGF1R, and PTK2 in adjacent nonneoplastic specimens (n = 20), primary HCC specimens (n = 20), and metastatic HCC specimens (n = 20) were shown. Scale bars, 200 µm (left), 50 µm (right). *p < 0.05, **p < 0.01, ***p < 0.001. Data were shown as Mean ± SD.

Given that BACH1 promoted HCC growth and metastasis through regulating IGF1R and PTK2 in HCC cells, we evaluated the correlation of BACH1 expression with IGF1R or PTK2 expression in primary human HCC specimens from two independent cohorts. IHC staining suggested that the levels of IGF1R and PTK2 positively correlated with the levels of BACH1 in both cohorts (Figure 6A-B). Kaplan-Meier analysis revealed that higher IGF1R or PTK2 levels in HCC patients correlated with worse OS and higher recurrence rates (Figure 6C-D). Both elevated IGF1R and PTK2 expression were related to increased tumor number, tumor encapsulation loss, microvascular invasion, less differentiation, and poor TNM staging (Table S5, S6). Noticeably, patients with either BACH1/IGF1R or BACH1/PTK2 coexpression exhibited the shortest OS and the highest recurrence rates (Figure 6E-F).

To further confirm the pro-metastatic properties of BACH1, IGF1R, and PTK2, we tested their expression in human metastatic HCC tissues. RT-qPCR and IHC examination suggested that BACH1, IGF1R, and PTK2 were elevated in primary HCC tissues compared to adjacent nonneoplastic tissues, while metastatic HCC tissues showed the highest levels of BACH1, IGF1R, and PTK2 (Figure 6G-I). Overall, these results supported the conclusions that BACH1 promoted HCC metastasis by regulating IGF1R and PTK2.

### IGF1R blockade combined with PTK2 inhibition enhances HCC therapeutic efficacy

**Figure 7:**
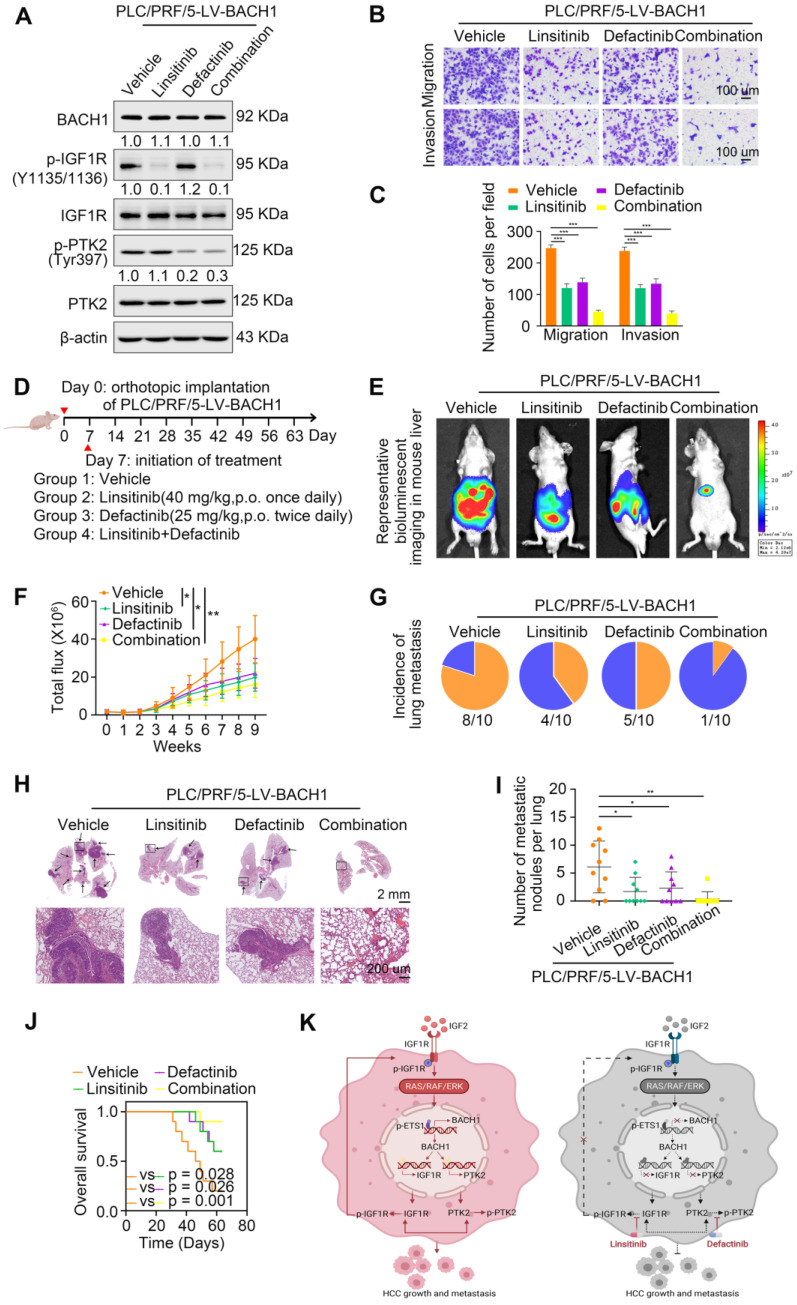
** IGF1R blockade combined with PTK2 inhibition suppresses BACH1-mediated HCC growth and metastasis. (A)** The protein levels of BACH1, p-IGF1R, IGF1R, p-PTK2, PTK2 in PLC/PRF/5-LV-BACH1 cells treated with linsitinib alone or defactinib alone or a combination of both. **(B-C)** Transwell assay was performed to analyze the migratory and invasive capacity of PLC/PRF/5-LV-BACH1 cells treated with linsitinib alone or defactinib alone or a combination of both. Scale bar, 100 µm. **(D)** Schematic diagram of drug treatment for nude mice. **(E-J)** HCC orthotopic xenograft models indicated that the combination of linsitinib and defactinib suppressed BACH1-mediated HCC growth and metastasis. The representative bioluminescent imaging in the liver (E), the bioluminescent signals of liver tumors (F), the occurrence of pulmonary metastasis (G), the representative H&E staining images of pulmonary tissues (H), the number of pulmonary metastasis lesions (I) from nude mice after orthotopic transplantation with PLC/PRF/5-LV-BACH1 cells treated with linsitinib alone or defactinib alone or a combination of both were shown. The OS of mice was analyzed in (J). Scale bars, 2 mm (upper), 200 µm (lower). *p < 0.05, **p < 0.01, ***p < 0.001. Data were shown as Mean ± SD. **(K)** A schematic diagram showing how BACH1 facilitates HCC growth and metastasis and the combination strategy for HCC. BACH1 upregulates IGF1R and PTK2 to promote HCC growth and metastasis. IGF2, the ligand of IGF1R, in turn upregulates BACH1 expression through the IGF1R-ERK1/2-ETS1 cascades, thus forming a positive feedback loop to stimulate HCC cells continuously. IGF1R blockade combined with PTK2 inhibition significantly suppresses BACH1-mediated HCC malignant progression.

Linsitinib (OSI-906), an orally bioavailable, selective, and potent dual IGF1R/INSR inhibitor, is being evaluated in clinical trials for multiple cancers 31, 32. Defactinib (VS-6063), an oral inhibitor of PTK2, has been explored in many preclinical and clinical studies for malignancies 21, 33. Since our findings suggested that IGF1R and PTK2 synergistically augmented the aggressiveness of HCC mediated by BACH1, we speculated that the combination of IGF1R inhibitors and PTK2 inhibitors might elicit an enhanced therapeutic efficacy for HCC. Thus, we treated the PLC/PRF/5-LV-BACH1 cells with either linsitinib alone or defactinib alone, or a combination of both (Figure 7A). As shown in Figure 7A, the levels of proteins BACH1, IGF1R and PTK2 were not affected by linsitinib and defactinib in the PLC/PRF/5-LV-BACH1 cells. This cell line was transfected with lentiviral vectors to stably overexpress BACH1, while BACH1 continuously transcriptionally activated IGF1R and PTK2 expression, which may be the reason why the levels of BACH1, IGF1R and PTK2 were not affected by these two inhibitors. To further verify the effects of these two inhibitors on the levels of BACH1, IGF1R and PTK2 in wild-type HCC cells, we treated the MHCC97H cells, which endogenously expressed BACH1, with either linsitinib alone or defactinib alone, or a combination of both (Figure S8). The results showed that linsitinib alone reduced the levels of BAHC1, IGF1R, p-IGF1R, PTK2, and p-PTK2. Defactinib alone reduced the phosphorylation of PTK2, but did not affect the levels of BACH1, IGF1R, p-IGF1R and PTK2. The combination of both reduced the levels of BACH1, IGF1R, PTK2, as well as the phosphorylation levels of IGF1R and PTK2. These results further confirmed the existence of IGF2-IGF1R-BACH1-IGF1R positive feedback loop in wild-type HCC cells, and linsitinib could disrupt this loop to reduce BACH1, IGF1R and PTK2 expression, as well as the phosphorylation of IGF1R and PTK2.

Transwell assays showed that both linsitinib and defactinib alone inhibited the migratory and invasive capacity of PLC/PRF/5-LV-BACH1 cells to some extent, whereas the combination of two inhibitors exhibited the most potent inhibition effect (Figure 7B-C). The same results were observed in orthotopic HCC metastatic models. Compared with treatment alone, the combination of linsitinib and defactinib significantly attenuated the growth of tumors in the liver, the occurrence of pulmonary metastasis, and the number of pulmonary metastasis lesions, thus improving nude mice OS (Figure 7E-J). These results demonstrated that IGF1R inhibitor linsitinib combined with PTK2 inhibitor defactinib prominently suppressed BACH1-induced HCC metastasis, providing a promising combination strategy for HCC therapy.

In addition, TAE226, a dual inhibitor of IGF1R and PTK2, attracted our attention. In a variety of malignancies, TAE226 induces cell cycle arrest, a decrease in cellular proliferation, and an increase in apoptosis in a time- and dose-dependent manner 34-36. TAE226 also inhibits tumor metastasis in breast cancer and Ewing sarcoma 37, 38. A recent study shows that combined TAE226 and sorafenib treatment reduce HCC growth *in vitro* and *in vivo* by affecting tumor-promoting gene expression and inducing epigenetic changes 25. These studies indicate that TAE226 has antitumor effects and has potential application in cancer treatment. Our results suggest that BACH1 facilitates HCC growth and metastasis by promoting IGF1R and PTK2 expression, so it will be of great significance to investigate whether TAE226 has a more potent effect in HCC with BACH1 overexpression. We treated the PLC/PRF/5-LV-BACH1 cells with either linsitinib alone or defactinib alone, or a combination of both, or TAE226 alone (Figure S9). The results showed that linsitinib alone and defactinib alone respectively reduced the phosphorylation of IGF1R and PTK2. The combination of both reduced the phosphorylation of IGF1R and PTK2. TAE226 alone also reduced the phosphorylation of IGF1R and PTK2, but its effect was weaker than that of linsitinib combined with defactinib (Figure S9A). Transwell assay showed that treatment with linsitinib alone or defactinib alone, or a combination of both, or TAE226 alone could significantly reduce the migration and invasion of PLC/PRF/5-LV-BACH1 cells. In addition, TAE226 showed a stronger inhibitory effect compared with linsitinib alone or defactinib alone. However, the inhibitory effect of TAE226 alone was weaker than that of linsitinib combined with defactinib, but this was not statistically significant (Figure S9B-C). These results indicated that the antitumor effect of TAE226 was comparable with that of linsitinib combined with defactinib in HCC. Linsitinib and defactinib are safe and show anti-tumor activity in several clinical trials of advanced solid tumors (https://clinicaltrials.gov). However, there are no clinical trials of TAE226, so more research is needed to explore its anti-tumor effects.

## Discussion

Transcription factors are commonly deregulated in cancer pathogenesis and are a major class of cancer cell dependencies 39. As a member of the bZIP transcription factor family, the function of BACH1 in multiple malignancies is well characterized, including breast cancer, lung cancer, colon cancer, etc. Nevertheless, the explicit role of BACH1 in HCC remains poorly defined. In this study, we demonstrated that BACH1 accelerated HCC growth and metastasis and BACH1 might serve as a promising predictive biomarker in HCC. In detail, overexpression of BACH1 facilitated HCC cells proliferation, migration and invasion, whereas knockdown of BACH1 strikingly inhibited HCC growth and metastasis. Moreover, BACH1 was upregulated in human primary HCC samples, and higher BACH1 levels in HCC patients correlated with worse OS and higher recurrence rates. Of note, BACH1 expression was prominently elevated in metastatic HCC specimens compared to primary HCC specimens, which further indicated the role of BACH1 in promoting metastasis.

The mechanisms underlying the tumorigenic and pro-metastatic role of BACH1 vary. On the one hand, BACH1 functions as a transcriptional repressor and inhibits metastasis suppressor Raf kinase inhibitory protein (RKIP) expression 40. On the other hand, BACH1 acts as a transcriptional activator, upregulating metabolism-related genes pyruvate dehydrogenase kinase (*PDK*), Hexokinase 2 (*HK2*), and *GAPDH* as well as pro-metastasis genes *MMP9* and *CXCR4* 9-11. However, the exact mechanism of BACH1 carcinogenesis and promoting metastasis in HCC has never been illustrated. Our data revealed that BACH1 upregulated cell motility-associated molecules, IGF1R and PTK2 to confer aggressiveness of HCC. IGF1R is a well-known proto-oncogene that plays vital roles in numerous malignant tumors, including HCC 18. IGF1R is prominently elevated in HCC tissues, and its expression is related to the poor prognosis of HCC patients 41. IGF2/IGF1R signaling alone provides a basal level of HCC cell motility, while in combination with HGF it supports the complete motogenic properties of HCC cells 28. IGF2/IGF1R cascades induce HCC cell migration, invasion, and lung colonization, which is mediated by matrix metalloproteinase-2 (MMP2) 19. A recent study shows that IGF1R-regulated constitutive activation of STAT3-midkine-STAT3 positive feedback loop drives HCC invasion 42. PTK2 is commonly overexpressed in cancer and regulates cell adhesion and migration 21. Increased levels of PTK2 and phosphorylated PTK2 Tyr397 are related to tumor staging, vascular invasion, and intrahepatic metastasis in HCC 43. PTK2 partly regulates HCC invasion and metastasis by upregulating and activating MMP2 and MMP9 43. These evidences indicate that IGF1R and PTK2 play crucial roles in the malignant progression of HCC. Consistently, our results suggested that direct upregulation of IGF1R and PTK2 contributed to BACH1-mediated HCC growth and metastasis.

BACH1 expression is regulated at translational or post-transcriptional levels in cancer cells 44. NRF2 promotes BACH1 accumulation by inhibiting the heme- and FBXO22-mediated degradation of BACH1 in lung cancer 12. HOXB8 interacts with BACH1 and activates BACH1 itself as well as its target genes transcription in colorectal cancer 45. However, the specific mechanism underlying BACH1 overexpression in HCC needs to be clarified. Our results demonstrated that IGF2 upregulated BACH1 in a dose-dependent manner. Inhibition of ERK1/2 pathway significantly abolished IGF2-induced BACH1 expression, whereas inhibition of PI3K/AKT pathway had no effect. The transcription factor ETS1 downstream of the ERK1/2 pathway activates BACH1 expression. In short, IGF2/IGF1R signaling upregulated BACH1 expression through the IGF2/IGF1R-ERK1/2-ETS1 cascades. In turn, overexpression of BACH1 provoked upregulation of IGF1R, which interacted with IGF2 and formed a positive feedback loop to amplify the tumorigenic and pro-metastatic effect of BACH1.

Our results showed that direct upregulation of IGF1R and PTK2 facilitated the tumorigenic and pro-metastatic properties of BACH1. To inhibit BACH1-mediated HCC growth and metastasis, we focused on inhibitors targeting IGF1R and PTK2. Linsitinib (OSI-906), one of IGF1R inhibitors, drew our attention due to its safety and antitumor activity 46. Due to the limited efficacy of monotherapy, the combination of linsitinib along with other drugs has been conducted in multiple basic research and clinical trials 47. Combining linsitinib with CSF-1R inhibitors significantly prolongs OS in the glioma mice model 48. The combination of linsitinib and CDK4/6 inhibitor synergically enhances antitumor efficacy in Ewing sarcoma 49. Linsitinib enhances the inhibition efficacy of sorafenib in HCC cells 50. The combination of linsitinib and EGFR inhibitor erlotinib is tolerable in patients with advanced solid tumors and induces durable objective responses in a minority of patients 51. Defactinib ( VS-6063), as an ATP-competitive PTK2 inhibitor, blocks tumor growth *in vitro* and *in vivo* 33. Defactinib shows an acceptable safety profile and modest anti-tumor activity in several clinical trials of advanced solid tumors 33. However, the efficacy of monotherapy is limited. Recently, multiple clinical trials for defactinib in combination with MEK/ERK inhibitors or anti-programmed cell death 1 (PD1) in patients with solid tumors are being evaluated 21. Based on the above evidences, we interrogated whether the combination of IGF1R inhibitor along with PTK2 inhibitor exerted synergistic effects in HCC. Our results demonstrated that compared with monotherapy, the combination of IGF1R inhibitor linsitinib and PTK2 inhibitor defactinib prominently enhanced anti-HCC efficacy *in vitro* and *in vivo*.

In summary, our study manifested that overexpression of BACH1 was a critical feature of HCC growth and metastasis. IGF2-induced BACH1 upregulated cancer progression-associated genes *IGF1R* and *PTK2*. IGF1R blockade combined with PTK2 inhibition enhanced HCC therapeutic efficacy. Our study provided a basis for developing the combination of IGF1R inhibitor and PTK2 inhibitor for HCC therapy.

## Supplementary Material

Supplementary methods, figures and tables.

## Figures and Tables

**Table 1:** Correlation between BACH1 expression and clinicopathological characteristics of HCCs in two independent cohorts of human HCC tissues

		Cohort I			Cohort II		
Clinicopathological variables		Tumor BACH1 expression		*P* Value	Tumor BACH1 expression		*P* Value
		Negative (n = 137)	Positive (n = 143)		Negative (n = 98)	Positive (n = 112)	
Age		51.82 (9.476)	52.54 (10.536)	0.552	52.70 (10.177)	52.71(10.932)	0.999
Sex	female	21	24	0.748	17	22	0.724
	male	116	119		81	90	
Serum AFP	≤ 20 ng/ml	24	25	1.000	28	22	0.146
	> 20 ng/ml	113	118		70	90	
Virus infection	HBV	90	103	0.603	75	83	0.893
	HCV	25	19		9	10	
	HBV + HCV	9	7		5	5	
	none	13	14		9	14	
Cirrrhosis	absent	35	44	0.335	28	29	0.756
	present	102	99		70	83	
Child-pugh score	Class A	123	113	0.014	74	84	1.000
	Class B	14	30		24	28	
Tumor number	single	110	81	**< 0.001**	70	54	**0.001**
	multiple	27	62		28	58	
Maximal tumor	≤ 5cm	96	66	**< 0.001**	52	47	0.128
size	> 5cm	41	77		46	65	
Tumor	absent	19	56	**< 0.001**	25	61	**< 0.001**
encapsulation	present	118	87		73	51	
Microvascular	absent	108	64	**< 0.001**	70	46	**< 0.001**
invasion	present	29	79		28	66	
Tumor	I-II	123	84	**< 0.001**	90	77	**< 0.001**
differentiation	III-Ⅳ	14	59		8	35	
TNM stage	I-II	132	91	**< 0.001**	94	76	**< 0.001**
	III	5	52		4	36	

Abbreviations: AFP: alpha fetal protein; BACH1: BTB and CNC homology 1; HCC: hepatocellular carcinoma; TNM: tumor-nodule-metastasis.

## Abbreviations

BACH1: BTB and CNC homology 1
CXCR4: C‐X‐C chemokine receptor type 4
CRC: colon cancer
ChIP: chromatin immunoprecipitation
EMT: epithelial-mesenchymal transition
HCC: hepatocellular carcinoma
HK2: hexokinase 2
H&E: hematoxylin and eosin
IGF1R: insulin-like growth factor 1 receptor
IGF2: insulin-like growth factor 2
IHC: immunohistochemical
MMP9: matrix metalloproteinase-9
MMP2: matrix metalloproteinase-2
mRNA: messenger RNA
OS: overall survival
PTK2: protein tyrosine kinase 2
PDK: pyruvate dehydrogenase kinase
PD1: programmed cell death 1
RT-qPCR: quantitative reverse transcription-polymerase chain reaction
RKIP: Raf kinase inhibitory protein
shRNA: short hairpin RNA
TNM: tumor-nodule-metastasis
VEGFR: vascular endothelial growth factor receptor
