# Supplementary material for: Overexpression of BACH1 mediated by IGF2 facilitates hepatocellular carcinoma growth and metastasis via IGF1R and PTK2
Source: Theranostics. 2022 Jan 1;12(3):1097–116. doi: 10.7150/thno.65775 (PMC8771560; doi:10.7150/thno.65775)
Supplement: Supplementary file 1 — Supplementary methods, figures and tables. [file thnov12p1097s1.pdf]

Supplementary materials

Methods

Cell lines and culture

PLC/PRF/5, HepG2, SNU423, Hep3B, SNU398, and SNU449 were purchased from the American Type Culture Collection (ATCC, MD, USA). Huh7 was purchased from the Stem Cell Bank, Chinese Academy of Sciences. MHCC97H, HCCLM3, and HCCLM6 were kindly provided by Dr. Tang ZY (Liver Cancer Institute, Zhongshan Hospital, Fudan University, Shanghai, China). All the cell lines were authenticated by short tandem repeats (STRs) DNA profiling. Additionally, all the cell lines were checked by the MycoAlert Mycoplasma detection kit. Cells were cultured in Dulbecco's Modified Eagle Medium (DMEM, GIBCO, CA, USA) containing 10% fetal bovine serum (FBS) (GIBCO, CA, USA) in 5% CO2 at 37 °C.

Patients and follow-up

This study was approved by the Ethics Committee of Tongji Medical College. All patients provided full consent for the study. Cohort I included 280 adult patients with HCC who underwent curative resection between 2003 and 2005 at the Tongji Hospital of Tongji Medical College (Wuhan, China). Cohort II included 210 adult patients with HCC who underwent curative resection between 2006 and 2008 at the Tongji Hospital of Tongji Medical College (Wuhan, China). A preoperative clinical diagnosis of HCC was based on the diagnostic criteria of the American Association for the Study of Liver Diseases. The inclusion criteria were as follows: (a) distinctive pathologic

diagnosis; (b) no preoperative anticancer treatment or distant metastases; (c) curative liver resection; and (d) complete clinicopathologic and follow-up data. The differentiation statuses were graded according to the method of Edmondson and Steine. The pTNM classification for HCC was based on The American Joint Committee on Cancer/International Union Against Cancer staging system (6th edition, 2002). Follow-up data were summarized at the end of December 2013 (Cohort I) and December 2016 (Cohort II, range 4-96 months) respectively. The patients were evaluated every 2-3 months during the first 2 years and every 3-6 months thereafter. All follow-up examinations were performed by physicians who were blinded to the study. During each check-up, the patients were monitored for tumor recurrence by measuring the serum AFP levels and by performing abdominal ultrasound examinations. A computed tomography and/or magnetic resonance imaging examination was performed every 3-6 months, together with a chest radiographic examination. The diagnostic criteria for HCC recurrence were the same as the preoperative criteria. The time to recurrence and overall survival were the primary endpoints. The time to recurrence was calculated from the date of resection to the date of a diagnosis with tumor recurrence. The overall survival was calculated from the date of resection to the date of death or of the last follow-up.

In addition, 20 normal liver tissues, 80 pairs of fresh HCC tissues and adjacent nontumor tissue samples and 20 pairs of fresh metastatic and matched primary HCC tissue and adjacent nontumor tissue samples were collected after surgical resection and were used for further investigations.

Construction of tissue microarrays and immunohistochemistry

HCC samples and the corresponding adjacent liver tissues were used to construct a tissue microarray (Shanghai Biochip Co., Ltd. Shanghai, China). IHC was performed on 4-μm-thick, routinely processed paraffin-embedded sections. Briefly, the tissue sections were deparaffinized after baking at 60 °C for 1 h. Endogenous peroxidase activity was blocked by 3% (vol/vol) hydrogen peroxide in methanol for 12 min and washes with phosphate-buffered saline (PBS). Then the slides were immersed in 0.01 mol/L citrate buffer solution (pH 6.0) and placed in a microwave oven for 30 min. After being washed with PBS, the sections were incubated with the primary antibody diluted in PBS containing 1% (wt/vol) bovine serum albumin at 4 °C overnight. Primary antibodies against BACH1 (Santa Cruz, sc-271211), IGF1R (Cell Signaling Technology, #3027), and PTK2 (Cell Signaling Technology, #3285) were used. Negative controls were performed by replacing the primary antibody with preimmune mouse serum. After being washed with PBS, the sections were treated with a peroxidase-conjugated second antibody (Santa Cruz) for 30 min at room temperature and then washed with PBS. Reaction product was visualized with diaminobenzidine for 2 min. Images were obtained under a light microscope (Olympus, Japan) equipped with a DP70 digital camera.

Analyses were performed by two independent observers who were blinded to the clinical outcome. The immunostaining intensity was scored on a scale of 0 to 3: 0 (negative), 1 (weak), 2 (medium) or 3 (strong). The percentage of positive cells was

evaluated on a scale of 0 to 4: 0 (negative), 1 (1%-25%), 2 (26%-50%), 3 (51%-75%), or 4 (76%-100%). The final immuno-activity scores were calculated by multiplying the above two scores, resulting in an overall score ranges from 0~12. Each case was ultimately considered "negative" if the final score ranges from 0~3, and "positive" if the final score ranges from 4~12 as described previously.

Western Blot Analysis

Proteins from lysed cells were fractionated by SDS-PAGE and transferred to nitrocellulose membranes. Nonspecific binding sites were blocked with 5% BSA in TBST (120 mM Tris–HCl (pH 7.4), 150 mM NaCl, and 0.05% Tween 20) for 2 h at room temperature. Blots were incubated with a specific antibody overnight at 4 °C. Western blotting of β-actin on the same membrane was used as a loading control. The membranes were then washed with TBST 3 times and incubated with an HRP-conjugated secondary antibody. Proteins were visualized using an ImmobilonTM Western Chemiluminescent HRP substrate (Millipore, MA, USA).

The primary antibodies used in western blotting were listed below.

Antibodies	Source
anti-BACH1	Santa Cruz, sc-271211
anti-IGF1R	Cell Signaling Technology, #3027
anti-PTK2	Cell Signaling Technology, #3285
anti-p-AKT(Ser473)	Cell signaling technology, #4060
anti-AKT	Proteintech, 10176-2-AP
anti-p-ERK1/2(T202/Y204)	Cell Signaling Technology, #4370

anti-ERK1/2	Cell Signaling Technology, #9102
anti-p-ETS1(Thr38)	Invitrogen, 44-1104G
anti-ETS1	Cell Signaling Technology, #14069
anti-IGF2	Abcam, ab9574
anti-pIGF1R(Tyr1135/1136)	Cell signaling technology, #3024
anti-pPTK2(Tyr397)	Cell signaling technology, #3283
anti-β-actin	Proteintech, 66009-1-Ig

Quantitative reverse-transcription PCR (RT-qPCR)

According to the manufacturer's protocol, total RNA was extracted with TRIzol Reagent (TaKaRa, Japan) and reverse-transcribed with the PrimeScript RT Reagent Kit (TaKaRa, Japan). Quantitative RT-PCR was performed on an ABI QuantStudio 3 (Applied Biosystems, MA, USA) with SYBR Premix ExTaq (TaKaRa, Japan). The cycling parameters were as follows: 95 °C for 5 s and 60 °C for 30 s for 40 cycles. The melting curve and the Ct value were analyzed. The $2^{-\Delta\Delta Ct}$ method was used to determine relative fold changes in target gene expression in cell lines, which was normalized to expression levels in corresponding control cells (defined as 1.0). The equation used was $2^{-\Delta\Delta Ct}$ ($\Delta Ct = \Delta Ct^{target} - \Delta Ct^{ACTB}$ $\Delta\Delta Ct = \Delta Ct^{expressing\ vector} - \Delta Ct^{control\ vector}$). When calculating relative expression levels in surgically extracted HCC samples, relative fold changes in target gene expression were normalized to expression values in normal liver tissues (defined as 1.0) using the following equation: $2^{-\Delta\Delta Ct}$ ($\Delta\Delta Ct = \Delta Ct^{tumor} - \Delta Ct^{nontumor}$). All experiments were performed in triplicate. The primer sequences were listed in Supplementary Table S7.

***In Vitro* Migration and Invasion Assays**

The migratory and invasive ability of HCC cells were evaluated using transwell inserts with an 8-μm pore size (Corning, NY, USA). DMEM supplemented with 10% FBS was added to the bottom chamber. Matrigel (50 μl, diluted 1:8 with DMEM, Corning, New York, USA) was coated on the top chambers and dried for invasion assay. 5×10^4 (migration assay) and 1×10^5 (invasion assay) cells were seeded in the top chamber in serum-free medium and were cultured in 5% CO2 at 37 °C for 24 h and 72 h, respectively. Cells that migrated or invaded to the lower surface of the membrane were fixed, stained, and imaged. The cell numbers from five fields per membrane of three inserts were used for statistical analysis. All experiments were performed in triplicate.

Construction of lentivirus and stable cell lines

Lentiviral vectors encoding shRNAs were generated using PLKO.1-TRC (Addgene) and designated as shBACH1, shIGF1R, shPTK2, shETS1 and shControl. ShControl is a non-target shRNA control. The vector "pLKO.1-puro Non-Target shRNA Control Plasmid DNA" (purchased from Sigma, SHC016) contains an shRNA insert that does not target any known genes from any species. The shRNA sequences can be found in Supplementary Table S8. Lentiviral vectors encoding human BACH1, IGF1R, PTK2 and IGF2 were constructed in pLV-puro or pLV-neo (Addgene) and designated as LV-BACH1, LV-IGF1R, LV-PTK2 and LV-IGF2. An empty vector was used as the

negative control and was designated as LV-control. The lentivirus and cell infection were produced according to the lentiviral vector protocol recommended by Addgene. Briefly, the lentiviral plasmid and packaging plasmids pMD2. G and psPAX2 (Addgene plasmid #12259 and #12260) were transfected into HEK-293T cells with transfection reagent (Lipofectamine®3000, Thermo Fisher Scientific) and OPTI-MEM media (Invitrogen, MA, USA). The lentiviruses were harvested twice on days 4 and 5. Viruses were filtered with a 0.45-μm filter and stored at -80 °C. For stable cell lines construction, HCC cells were transfected with lentivirus at a multiplicity of infection (MOI) of 10-30 for 12-24 h. 72 h after infection, HCC cells were selected with 2.5 μg/ml puromycin (OriGene) for 2 weeks. The stable cell lines were confirmed by qRT-PCR and western blotting.

Luciferase reporter assays

The Dual-Luciferase Reporter Assay (Promega, CA, USA) was used to detect luciferase activity according to the manufacturer's instructions. In brief, the cells transfected with plasmids were lysed and the lysates were centrifuged at maximum speed for 1 min. Relative luciferase activity was determined using a ModulusTM TD20/20 Luminometer (Turner Biosystems, CA, USA) and was normalized to Renilla luciferase activity.

Plasmid construction

Plasmid construction was performed according to standard procedures. The primers

were shown in Supplementary Table S7. For example, the *BACH1* gene complete CDS construct, pCMV-BACH1, was generated by using cDNA from human PBMCs. It was generated with forward and reversed primers incorporating EcoRI and BamHI sites at the 5' and 3'-ends, respectively. The polymerase chain reaction (PCR) product was cloned into the EcoRI and BamHI sites of the pCMV-Tag2B vector. The *IGF1R* promoter construct, (-2128/+70) *IGF1R*, was generated from human genomic DNA. This construct corresponds to sequence from -2128 to +70 (relative to the transcriptional start site) of the 5'-flanking region of human *IGF1R* gene. It was generated with forward and reverse primers incorporating KpnI and MluI sites at the 5' and 3'-ends, respectively. The polymerase chain reaction (PCR) product was cloned into the KpnI- and MluI sites of the pGL3-Basic vector (Promega, CA, USA). The 5'-flanking deletion constructs of the *IGF1R* promoter, (-1720/+70) IGF1R, (-572/+70) IGF1R were similarly generated using the (-2128/+70) IGF1R construct as the template. The BACH1 binding sites in the *IGF1R* promoter were mutated using the QuikChange II Site-Directed Mutagenesis Kit (Stratagene, CA, USA). The constructs were confirmed by DNA sequencing. Other promoter constructs were cloned in the same manner.

Transient transfection

The cells were plated at a density of 1×10^5 cells/well in a 24-well plate. After 12-24 h, the cells were co-transfected with 0.6 μg of expression vector plasmids, 0.18 μg of promoter reporter plasmids, and 0.02 μg of pRL-TK plasmids using Lipofectamine

2000 (Invitrogen, MA, USA) according to the manufacturer's instructions. After 6 h of transfection, the cells were washed and allowed to recover overnight in fresh medium supplemented with 1% FBS for 48 h. Serum-starved cells were used for the assay.

Chromatin immunoprecipitation Assay (ChIP)

Cells were immersed in 1% formaldehyde for 10 min at 37 °C to stimulate cross-linking. Then, glycine was used to quench the formaldehyde after cross-linking to stop formaldehyde fixation. After washing with PBS, the cells were resuspended in lysis buffer (1 mM PMSF, 1% SDS, 10 mM EDTA and 50 mM Tris (pH 8.1) – total volume 300 µl). Sonication was then performed to produce fragmented DNA. A slurry of protein G-Sepharose and herring sperm DNA (Sigma-Aldrich) was used to clear the supernatant. The recovered supernatant was then subjected to a 2-hour incubation period with specific antibodies or an isotype control IgG in the presence of protein G-Sepharose beads and herring sperm DNA, followed by antibody denaturation with 1% SDS in lysis buffer. Precipitated DNA was extracted from the beads by immersing them in a 1.1 M $NaHCO_3$ solution and 1% SDS solution at 65 °C for 6 h. Immunoprecipitated DNA was retrieved from the beads by immersion in 1% SDS and a 1.1 M $NaHCO_3$ solution at 65 °C for 6 h. The DNA was then purified using a PCR Purification Kit (Qiagen, Germany). The primers were shown in Supplementary Table S7.

For ChIP assays of tissues, cells were first separated from six pairs of fresh frozen

HCC tissues and normal liver tissues collected after surgical resection. In detail, surgically extracted tumor tissues were first washed by 1 × cold PBS, 5 min, for three times and added to medium supplemented with antibiotics and antifungal agents. Use a clean razor blade to cut a pie of tissue (around 5 mm^3) into small piece (typical 1 mm^3 or smaller). Then, digestion the tissues with DNase I (20 mg/mL; Sigma-Aldrich) and collagenase (1.5 mg/mL; Sigma-Aldrich) and placed on a table concentrator, 37 °C, for 1 h. At the end of the hour, we filtered the dissociated cells through 70 μm-pore filters rinsed with fresh media. The 1 × red cell lysis was added to the tissues and incubated for 5 min to lysis the red blood cell, followed by another rinse. The dissociated cells were crosslinked using 1% formaldehyde for 10 min at 37 °C. After cell lysis, the DNA was fragmented by sonication. ChIP grade antibody or IgG (negative control) was used to immunoprecipitate the fragment DNA. Then, qRT-PCR was used to amplify the corresponding binding site on the promoters.

The antibodies used in ChIP were listed below.

Antibodies	Source
anti-BACH1	R&D System, AF5776
anti-RNA Pol II	Santa Cruz, sc-47701
IgG (normal mouse IgG)	Santa Cruz, sc-2025
anti-ETS1	Cell signaling technology, #14069

Cell Counting Kit-8 (CCK8) assay

For cell proliferation studies, HCC cells were seeded into 96-well plates (5000 cells/well). Six wells of each group were detected every day. The cells were incubated

into 100 μl of fresh medium containing 10 μl CCK8 at 37 °C for 2 h, and then the medium was replaced by 100 μl of DMSO and shaken at room temperature for 10 min. The absorbance was measured at 450 nm.

Colony formation assay

For colony formation assays, HCC cells were seeded into 35 mm dishes (500 cells/dish). Then the cells were incubated at 37 °C in 5% CO2 for 2 weeks. Subsequently, the medium was removed. The cells were fixed with 4% paraformaldehyde, stained with 0.1% crystal violet and imaged with light microscope (Olympus, Japan). Only positive colonies (diameter > 40 um) in the dishes were counted and compared.

***In vivo* tumor growth in the xenograft model**

All animal experiments were approved by the Committee on the Tongji Hospital of Tongji Medical College, Huazhong University of Science and Technology. BALB/C nude mice (male, five weeks old) were housed and cared according to the institutional guidelines for animal care. For the *in vivo* growth assay, suspended treated cells were subcutaneously injected into the flank of each mouse (ten mice per group, 1×10^6 cells in 150 μl of PBS per mouse). The mice were weighed and the tumor size was measured using vernier calipers. The tumor volume was calculated using the following equation: $V (mm^3) = 0.5 \times L (mm) \times W^2 (mm^2)$. After four weeks, all mice were sacrificed. Then, tumor weight was measured. The tumors were then embedded

in paraffin and prepared for H&E staining.

Supplementary Figures

Figure S1

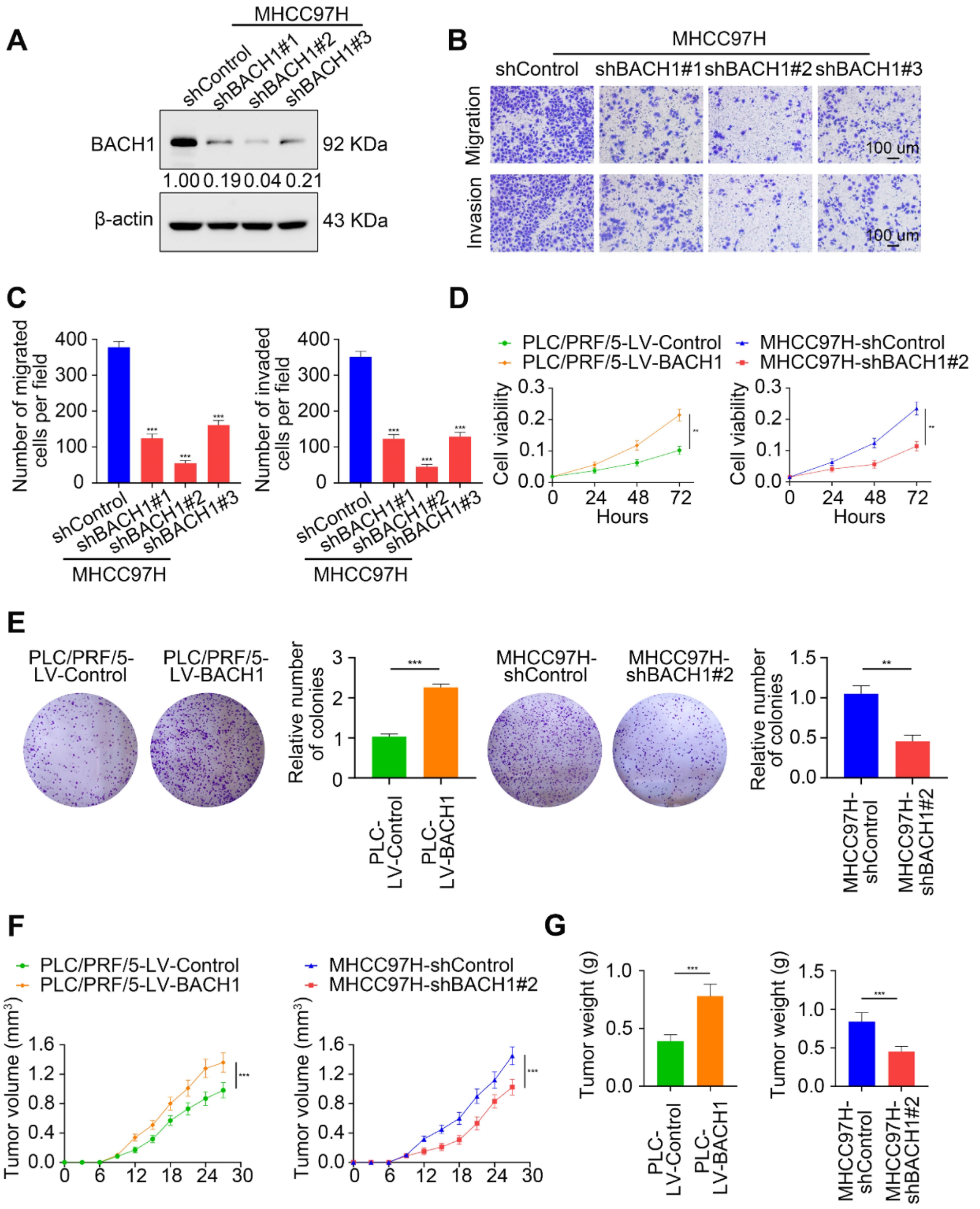

Figure S1.

(A) MHCC97H cells were treated with three different shRNAs targeting BACH1

(shBACH#1, shBACH1#2, shBACH1#3). Western blot verifying BACH1 knockdown effect in MHCC97H cells.

(B-C) The migratory and invasive capacity of the indicated MHCC97H cells were detected by transwell assay. Scale bar, 100 μm.

(D) Cell Counting Kit-8 (CCK8) assay assessing the cell proliferation of the BACH1-overexpressing PLC/PRF/5 cells and BAHC1-knockdown MHCC-97H cells.

(E) Colony formation assay showing the proliferation of the indicated HCC cells. The representative photos were shown and the cell numbers were quantified.

(F-G) Tumor growth of the indicated HCC cells was assessed by subcutaneous xenograft tumor models. The tumor volume and weight were shown in (F) and (G), respectively. n = 10 in each group.

**p < 0.01, ***p < 0.001. Data were shown as Mean ± SD.

Figure S2

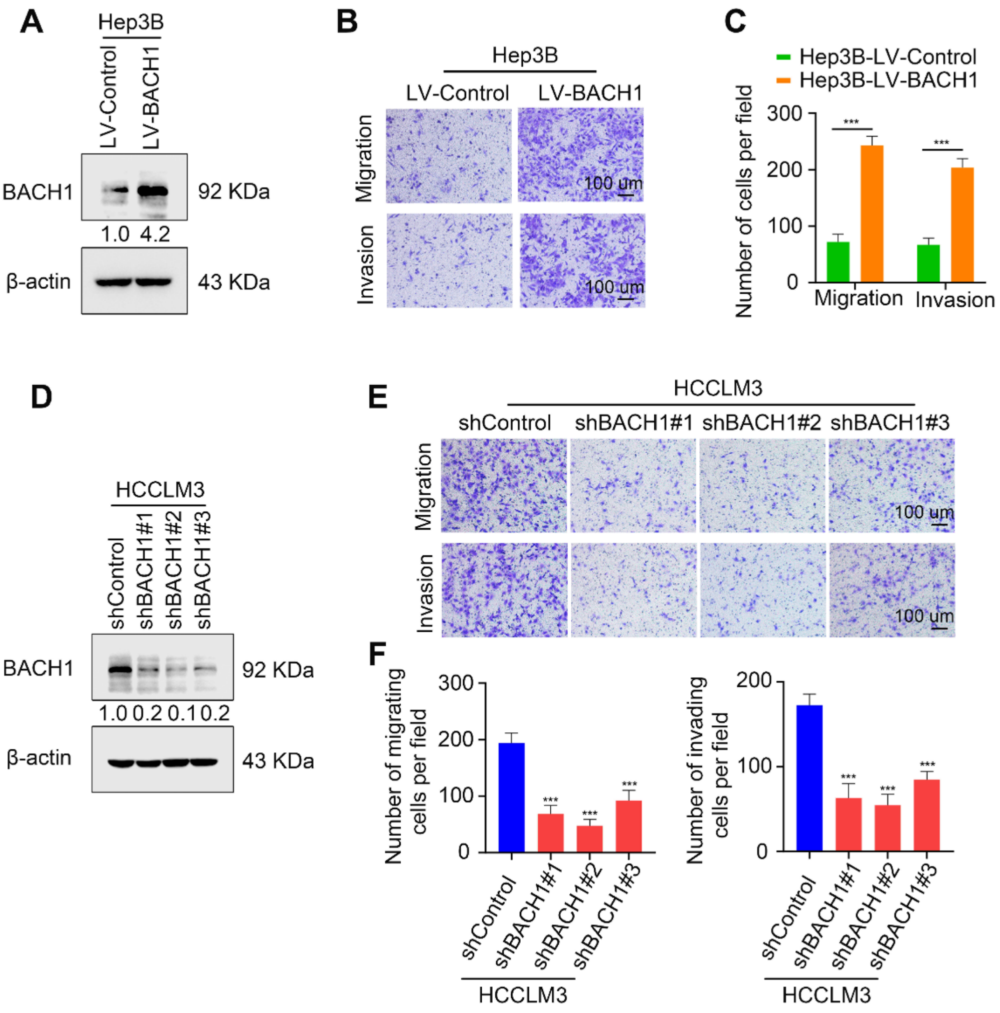

Figure S2. Effects of overexpression or knockdown of BACH1 on migration and invasion of Hep3B and HCCLM3 cells.

(A) Western blot verifying BACH1 overexpression effect in Hep3B cells.

(B-C) The migration and invasion capacity of BACH1-overexpressing Hep3B cells were detected by transwell assay. Scale bar, 100 μm.

(D) Western blot verifying BACH1 knockdown effect in HCCLM3 cells.

(E-F) The migration and invasion capacity of BACH1-knockdown HCCLM3 cells

were detected by transwell assay. Scale bar, 100 μm.

***$p < 0.001$. Data were shown as Mean ± SD.

Figure S3

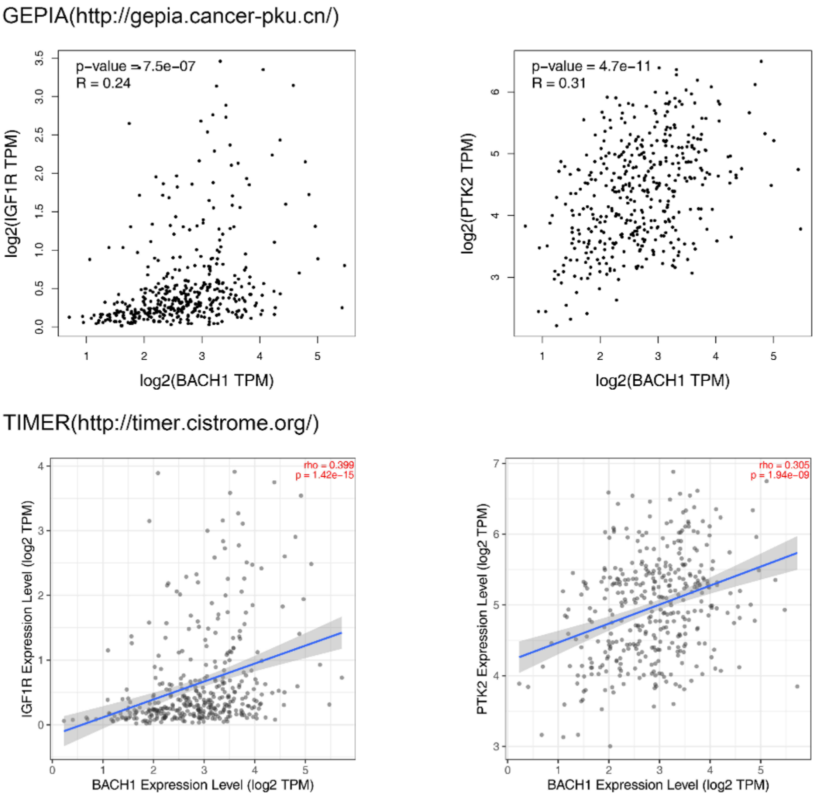

Figure S3. The correlation between *BACH1* expression and *IGF1R* or *PTK2* expression in TCGA-LIHC database.

IGF1R and PTK2 expression positively correlated with BACH1 expression in TCGA-LIHC database (http://gepia.cancer-pku.cn/; http://timer.cistrome.org/).

Figure S4

IGF1R Promoter (-1959 ~ +97)

>NC_000015.10:98646580-98648635 Homo sapiens chromosome 15, GRCh38.p13 Primary Assembly

-1959 AAACTGAAACTCTTTATTTAAAAATCAAGCTGAATTTCAGTTAAACAAAACCATCCCATCATATGAATAACTTTCTTAGGTAAAACAAGGTT

-1867 TATTTTCTTTCTATACAACTGACTCTGAATTGAGCTAGAAATTTCCAAGGAGGAAAATGATCTAGGAAACAACTTTAGAAAAAAAGGGCTA

BACH1 binding site 2

-1776 AGTTTCCGTTATGATAGCTTTTGACTTGTTTTCAGCTCTTAAAAAATTATTTACGAACGATGGATACACGTTCTAATGCAGAAGTATTTTAGA

-1683 ATTAGAGAGTAAAAGAAACCTACTACCTTCCTTTACATCAGGTCCCTTCTACCATCCTACCCGATTGTTTGAGACAACCACTTCTTATCTCG

-1591 ACAATTCACAACTCTTTTATTAGCTATCTTAAAAAAATTTATTACTGGCATCAATTAGCCTGAGTCATGAAACCGGACCACATTAAGGGCGA

BACH1 binding site 1

-1499 CACATGGTCCAATCACTGTTTGTAAAAGTCCACGTATTTCAAACTCCTCTCTCCTGCCACTGCTGGGCTGTTTCCCTCTTTGAGGACCTGG

-1408 TCTCCGCAGCATTTATTCATTAGATGGCAGTCCTAGGGGAGTCTCGCTTTGGGGAAACCTCTCCTCCTGCACATTCAAGAAAACAACCGCG

-1317 GAGACTTAGGGTCGGTACTGGTTTCCAGTCACTTACGTAGCAAACGAAGCAAGAGGAACGTGCCTGGGAGGACCCGAGACAGGTGCGG

-1229 GTGGGTTTCCGCAGTAGCCGCTGATCCCGAGTGCATGCGGCGTGTTCCCGGGTCGGGACCGCGGCCAAGGGAGGCTTCCCGGCCCCAGC

-1140 CTCCACCCCCTCCTCGGCGCCCCGGGACCCGGACACGCCCCCCGAGCTTCGGAGACCCGCAGCGTGCACGCGCCCCGGCCGCTCCCCGC

-1051 AGCCGCCCACGTGGTGGAGCCCTGAGCTGCGCGAGGCCGCGGAGAGCGCTCAGGGCGGGCGGCTGGTCCGGGAGGCCACGCCAGCGC

-964 GACCCAGCCGAGTCGGCCCCCAGCCCGGGCCCCCACATTTCCTCCCCCGGAGGGAGGGAGGCGACTCTCCGCGGGCTGCCCTCCCCAGC

-875 GCCCGCCGCGCCCTCTGGCGGCCGCCGCGGGGACGCGCCCGGGGCACGCGGCGCTGCCTGTCTGGGCCCCCCTTCCGGGGCGCGGGGCC

-786 CGCGAGGGGCGGCGGGGTCCTCTCTCCTCGAGCCACTCTGGGCCGAGCCACACGGGCCGCGCCCTTCCCCCTCCGCTCCCCCTGAGCCCC

-696 CAAACTCCGGGCTCCACGGTCGCAACGCCGCGGGCACCCCAGCCTGGCGTGAAAGTGCCCGGCGTAGTAGCCTGGGGGGGGGGTCCCCT

-607 TTCTCCCAGGTGCGCCCCTTCCGCCACGTTCGGGCTTTCCAGTACGCAGCGAAAAAAATGCCGCATGCACGCATTTATTTATTTGCAACAG

-515 CTGCAAGAAACAATGAAGCTTTTCAAGAACCGGGGAAACGCGCTTTCCAGCCGCGCTGTTGTTGTTTTCAATGAACCTCTCCCAGCCCCG

-425 CACTCCCCGCCCACCCCTCCCCTCTCCTGCCCACCCCTCCCCTGCCTAGCCTTTCCCTGGCTACCCACCCCTGCCCCGCCGAGACCGGACC

-334 GGCGGCGGGGGCATTGTTTTTGGAGTCGGGCGGGAGGGGAGGGCGCGTGCGGGGTGGCCGGCGCAGTGCGGTGGGGGCGGGAGCGGGT

-246 GGGCACGCGCGCGTGTCTCTGTGTGCGCGCGGGAGGCGGTGGGGCGGGAGATGGGGGCGGCGCCTCGCAGTCTCGCGCCCCACGCCCGG

-157 GCTCCGCTCCGCACGTCTTGGGGAACCGGGCTCCGGTTTTTTGCGCGCGCCGGCCTGGGCCGGGCCCTCGGCGCGCCGCTGCTGCGGCGG

-67 TGGCCGCTCGAGTGTGCGAGCGGGCGCGTGTGCGCGGGCCAGGGCGCGCGCGCGCGCGCGAGCCCCCAGTGTGTGGCAGCGGCGGCGGC

Transcription starting sites (nucleotide +1)

+23 GGCGCGGCGAGGCTGGGGCTCTTGTTTACCAGCATTAACTCCGCTGAGCGGAAAAAAAAAGGGAAAAAACCCGAG

Figure S4. BACH1 binding sites within the promoter regions of *IGF1R*.

The sequences highlighted in yellow represent the two binding sites of BACH1 on the *IGF1R* promoter, and the arrow represents the transcription initiation sites.

Figure S5

PTK2 Promoter (-2420 ~ +112)

>NC_000008.11:c141004499-141001968 Homo sapiens chromosome 8, GRCh38.p13 Primary Assembly

-2420 CAAATTTATAAAATGGAGGTGCCGATAATAGCGTCTACATCACAGGGTTGCTATGAGGTCTGGAGACAGCGTATTCAGGTTCTGACCCACA

-2329 GAGACTGAGAGAAGTCAGCCTTCCTTGACTCTGTGCTCTGGTGGTCCTTTGCATACCCCTCCATCATCATGCAAATTATTAGTAATCGATAA

BACH1 binding site 3

-2237 AGCAATGCTATAGTCGATCAATAACACTTATATATGCTAAGCACCCTGCTGTTTTATGAGAATGGTTTGTTTCCTCTACAACCTAATTTTTTTT

-2143 TTTGAGATGGAGTCTTGCTCTGTCACCCAGGCTGGAGTGCAGTGGCGCCATCTCCACCCACTGCAAGCTCTGCCTCCCGGGTTCACGCCG

-2053 TTCTCCTGCCTCAGCCTCCCGAATAGCCGGGACTACAGGCGCCCGCCACCACGCTGGCTAATTTTTTTGTATTTTTAGTAGAGACGGGGTT

BACH1 binding site 2

-1962 TCACCTTGTTAGCCAGGATGGTCTCAATCTCCTGACCTCATGATCCACCCGCCTTGGCCTCCCACAGTGCTGGGATTACAGGTGTGAGCCA

-1871 CCGTGCCCGGCCTCTCTACAGCCTAATTCTAATGTACATCCTTTAGAGCCTCCATTTCACAGATGAGAAATTGAGGCTCAGAGACATTATAC

-1779 AACCTTCACAGGTTTATTGGTGAATGGTTAAACTTAAGCCTGGGTCTGTGCCAATGTTCATCTTACTCCTAAACCTGAGTTCTCAGGCACC

BACH1 binding site 1

-1688 ACAAAGTGTGCCCAAGAGGTAAGACAAGCCACTGTTTCCTAGGTTTGTCCCTTTTCCCGTTTGTATCTCTGTTTGCCCTTCTCCTTTGCATC

-1596 ACTTCTTCCCGCCGTTTTCTGATTTCTTCTTTTACTAAGCATAATAATAACCAAAGCTGCCCTTCAAGAACGTCAATTTCAAATGTATCGTGG

-1503 CAGAGTCCAGGAAACAATATTGTGTAACACACTACCCTAAAACCTAAAGGCTTAAAACCACATGTATTTTATTGGCTCACAATTCTGTGGT

-1412 TTGGCAATTTGGGTTGGATTCAGCTGGGCAGTTCTTCCATCAGTCTCTCCAGGTCTTATGTATGCTTCACAGGGGCTGGGTGGTCTAGTACA

-1320 GCTCACTCACATGTCTGGAGGTGGCACCTTGTTGGCCAGGGTGCCTCAGTTCTCTTCCACACGGCCTCCTCGTGAGGCTAGCTTGGGCTT

-1230 AAGGGGGGAAAATGAAAGCAGCATGGCCTCTTGAGGCCTAGGCTTGGAATTTGTACAATGTCACTTGTGACACATCCCATTGGTCAAAGC

-1140 AAGTTACAAGATCCCATCCACATTCAAGGGGTGAATAAATTCCACCTCTTCTTGGGAAAAGTGTCAAAGAATTGATGGCCATTTTTAGTCT

-1049 ACTATGCTAAGTGCAGCCCCTTCCCCCAAAGTTATCCTTAATGCAGTCCCTTTGTGCCTCCACTCTCCTCTTTTAATTCCCATGCTTTTCTTAT

-955 CCTTTTAATAATAAGCTCTCACTTCACATCACCCAAAGCTTCGTTCATTCACTTGACAAATACTTACTGTGTGCCAGGACCTATTAGTGAACA

-862 AAACAAAGCCCGTGCTCTAATGAATCTAAGATTCTAGTGGAGAGAGCGGACAATCAAGAAATAAACAAGAACACAGTGTTGGGCTATGGT

-772 GAACAATAGGTGCTTTGGAGAACAACAGAGCAGACTAAGTGCGGACTGTGTGTGAGGATAGCAGGGCAGTGGGTGGTTCCTATTATATGA

-682 AGTGGTCGGCTGTATCAATGAGGTGATATATAAGCAGGGTATTGAACGAAGTGAGGAGCAAGAAGTTCCAGGCAGAGCAAATAGCACGCG

-592 CAAAGGCCTGAGGCTGGCATGGAGAGGCAATTCCTTACAATTTCATATGTAGTAAAAAGTAACAAAACCCAGAAAAACACCAACCTTAATA

-501 CATCTCATTAAAGAACAATACAACCCAAACATTTAAATATGGTAAAATCTTGATAAGCAAAAGCTTTTTATTTTACTTCATTTTTACTTTTATA

-407 AAAGCTCCTAATGCAATGACGTGTGTCACCTCTCTGGGCTTCAGGGTGTTCACCTTTCAAAAGGCCTCGGTGCCTGTCTGTCCTGCTTCCTG

-315 GCTTGTGGTTAGCATCAAAATGACACCATAAAAATGAAAGTTGCAGCCTTGAGGGTTCTTCTGGTTTCATTTCCGACCACAAGGTTGCCAG

-224 AGTGACTTTTTTTTTTTTTTAAATAACACAAATCAGTTATCACTTCCTGCTTAAAGCCCTCCAAAGGTTCCCTGTTGCCTAAAGAATAAACTC

-131 CAGGCACCTAAGCAAGCCCCACGCAGCCGTCTGCATTTTTCAATCCCTCCAACCTCGCCTTTTGCTATTTCCCCCACCCCACCTACGGCACG

-39 TTCCGGTCATAACCAACTAGCTCCTCGCTGAAACTGGCTCACACCGCGGAAGCCCGGGTCGGTGTCGGGGCGAGCCTTCCCTCCTTTTCCTG

Transcription starting sites (nucleotide +1)

+54 GAGCCCGTCTCAGGTCTGTAGCCCTCGGGAGGGATTGCAGGGCTCGTTCCCTGCTGGCG

Figure S5. BACH1 binding sites within the promoter regions of *PTK2*.

The sequences highlighted in yellow represent the three binding sites of BACH1 on the *PTK2* promoter, and the arrow represents the transcription initiation sites.

Figure S6

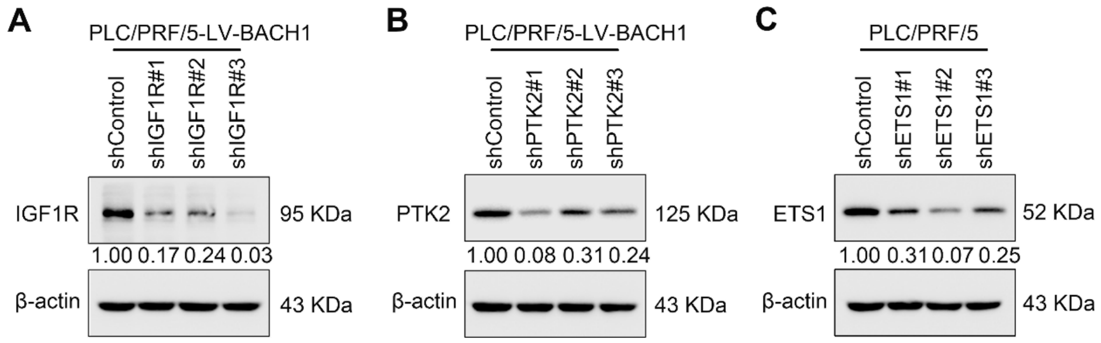

Figure S6. Western blot verifying IGF1R, PTK2 and ETS1 knockdown effect in PLC/PRF/5-BACH1 cells.

(A-C) PLC/PRF/5-LV-BACH1 cells were treated with shRNAs targeting IGF1R (A), and PTK2 (B). PLC/PRF/5 cells were treated with shRNAs targeting ETS1 (C). Western blot verifying IGF1R, PTK2 and ETS1 knockdown effect in the indicated cells.

Figure S7

BACH1 Promoter (-1908 ~ +127)

>NC_000021.9:29297014-29299048 Homo sapiens chromosome 21, GRCh38.p13 Primary Assembly

-1908 TAGTAGAAACGGGGTTTCACCATGTTGGGGTTTAGTAGAAACGGGGTTTCACCATGTCGATCTCCTGACCTCGTGATCCGCCCGCCTTGGC

-1817 CTTTGAAAGTGCTGGGATTACAGGCATGAGCCACTGCTCCCGGCCGGAATCTGCATTTTAACAAGCAAACCAAATATTCTTCCCCATAATG

ELK1 binding site 2

-1726 AAGCTTGAGAGTCACTCACTTGACTTGATACTCTTAGCACATCTGTATACCTTTGTGAGAACTTCTCAAATAATTAAACCTATCAGGGCATA

-1634 CATCATAATTTTCAGGCTAGAAAATGTCACTGCTATTATCTAAAGAAGCAAGAGTTCCTCCCAAAGAGGAGGGTCTGATCTTCCCCTAGAA

-1543 AAAACTGCACAGGTGTAGAAAAAACTGCATAGGTGTAGAAAAAAGACACTGCAATTCCACTGGCTCAAGGTGGAAGGAGAATAAAGCC

-1455 TCGGACACTCCCATGTGTCTGCAAGAACTTCAATCCTTCTTTCATGGTGTATTTCCAAGTGTCCTTTCCTCCTGTTTCACTAAATATCTGCAC

ETS1 binding site 3

-1362 TCTCACCATGAACTGAGGCAGCCTGGGATGCGAGGTACCTCCTACCTCTGGACCCTATCTAGACTGCTGGGTTCCACTCTTCCCAATCAAG

-1271 ACCCAATGCAAATGTCACCTGCTCCAGGAAGCCTCCCCTAATTCTAGGTCATAATGTATCACTTCACCCCTCGCACTCTCATCCAATTTCAC

ELK1 binding site 1

-1179 TTGTGTGATTTTCTCATGTATTTTGGATGTAGCTCAAACGTCCCCAAGTCCCACAGCCTGTGGGTGCAAATGCAGTCATGTTAGTCGTAATA

-1087 CCTGAATTATTATCTTGGTATCCTTTTTGCCCCGGGAACTCCAGGGCTGTGTTCTTGTTGCTGGCTCCAAAGCTAACAATAAAATCCCTTCA

-995 CACAAATCGTCCTTTGGCTTCAGACCTCAAGGTCCGTGTCATCTCCGCAGGAACCCTCGTGGGGTTGGCGTGCCCAGTCCCCTCCGCTGCG

-904 CGTGCATCCCGCTCCGCTGACAGCCAGTGCTTTGGTTCTCCAGAGGTGTTAAATGGCTTGGGAACATAATTTCAAAACGGACAGTTGGATA

HIF1A binding site

-813 AACACCAGAATAAGCACCAGAAGATGACAGCGGAACCAAGGCTCGAGATGGGCCGGCTGACACCACTGGGCGCTGAACCTGGCTGGAAG

-724 CTTCCTGGCAGCGACAGCGAAAAGGGAACACCTGTCGTGGGCGCCCTTGTTTCACTGTCAGTAAGAAGTAGAGCAACTGCTAGACGCGCT

ETS1 binding site 2

-634 CCACAAAACCCTGGACGCACTTCATACACACCCCCACCCCAGCAAGCTCAAGTACCCTGTGAACCTGGACGCCCACCACCCCCAGGGCAG

-544 GCAGCGAGGAGCTCTTCAAGGGGTAAAGCGACCCCTGTGGGGCCAGCGCCCGTCCTCTCTCCTGCTGTTCGCGGGCACCCGGGCACGCG

-455 GCGACTCGGCTGAATTAGGGCGTCCTGCAGCTCCCGAGGCGAGGAGGCGCTTGGCCTTTCCGTTCCGCCCTCAGAAGGGAGAGGAGAGG

-366 TGGTCCTCGAGTGGGAGTCAGTTCAGGATGCGGGCTGTGCCACGCGCGGAGGTCCGGGGACCCCACCGCCGCCTCCTCGAGGCCCGCCCC

AP-1 binding site

-276 GCCCCTTACGACCCGTCCCTGCAACCCCCCGCGTGGGCTTCCGGCCGCGGCGACCCCTGCCCCGCGCTATTGCGCGCGCTCGTTCGCTGAC

ETS1 binding site 1

-185 AGAGGCTTCGAGGGCGGCAGGGCGGGGCTTCGGCGCCGGGGGCGGGGCCTTCAGCGGGCGGGCGGGGTTTTGGCGCCGCGAGGTGGCG

SP-1 binding site

-97 GCAGCAGAGCGGCGCAGAAGGGAGGGGGCGGCTGGCCGCGCGGAAGGAGTGAGTCACCTGACCGCTGCCCTCGCCGCCCGCCGGGCGCT

-8 CTCGCTTCAGTCAGTCGGGCCGCGCCGCGCCTCAGCTCTGGTGAGTGGCTCGGCCGTCCCGCCGGCCCTTCTCCGGGAGGGTTGGCGCGGTC

Transcription starting sites (nucleotide +1)

+85 AGGGCCGCGGGCCTGTGAGGGGAGGCCGGCGGACAGGTCCAGT

Figure S7. Transcription factors binding sites within the promoter regions of *BACH1*.

The sequences highlighted in blue represent the two binding sites of ELK1 on the

BACH1 promoter. The yellow highlighted sequences represent the three binding sites of ETS1 on the *BACH1* promoter. The sequences highlighted in green represent the binding site of HIF1A on the *BACH1* promoter. The orange highlighted sequences represent the binding site of AP-1 on the *BACH1* promoter. The purple highlighted sequences represent the binding sequence of SP-1 on the *BACH1* promoter. The arrows represent transcription start sites.

Figure S8

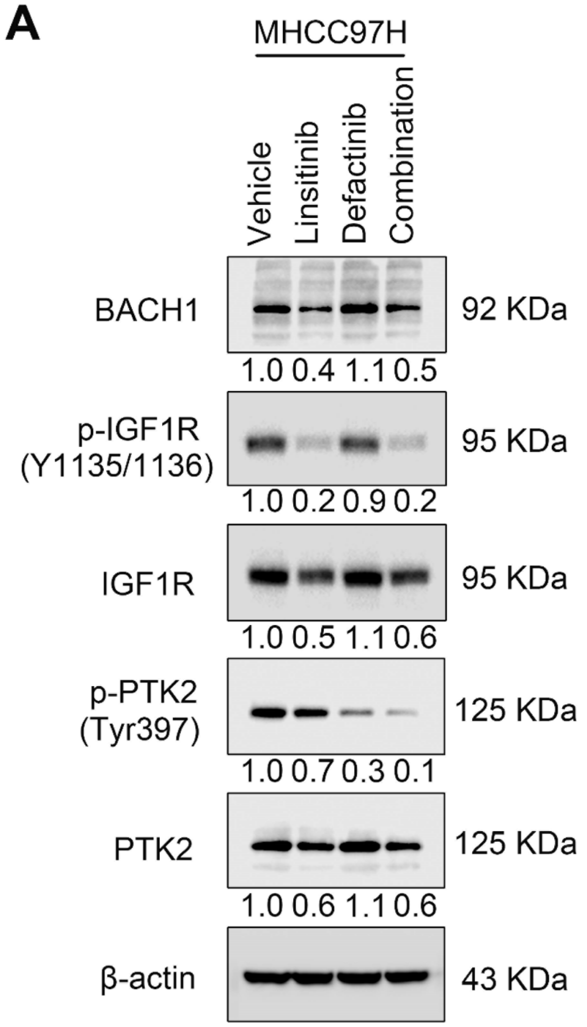

Figure S8. Western blot verifying the effects of linsitinib alone or defactinib alone, or a combination of both on BACH1, IGF1R, p-IGF1R, p-PTK2 and PTK2 expression in wild-type MHCC97H cells.

(A) The levels of BACH1, IGF1R, p-IGF1R, PTK2 and p-PTK2 in the MHCC97H cells treated with linsitinib alone or defactinib alone or a combination of both.

Figure S9

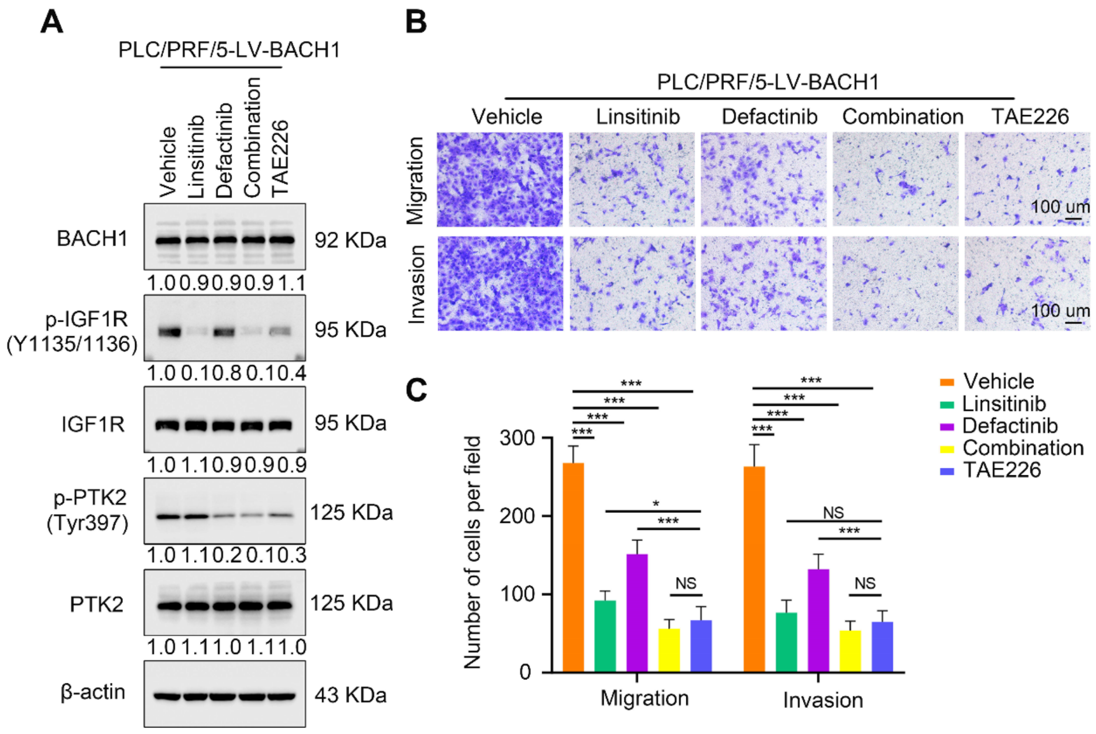

Figure S9. Effects of TAE226 on migration and invasion of PLC/PRF/5-LV-BACH1 cells.

(A) The levels of BACH1, p-IGF1R, IGF1R, p-PTK2 and PTK2 in the PLC/PRF/5-LV-BACH1 cells treated with linsitinib alone or defactinib alone or a combination of both, or TAE226 alone.

(B-C) Transwell assay of migration and invasion of PLC/PRF/5-LV-BACH1 cells treated with indicated inhibitors. Scale bar, 100 μm.

*p < 0.05, ***p < 0.001, NS: no statistical difference. Data were shown as Mean ± SD.

Supplementary Table S1 Univariate and Multivariate Analysis of Factors Associated with Time to Recurrence and Overall Survival in Cohort I HCC Patients (n = 280)

	Time To Recurrence		Overall Survival	
Clinical Variables	HR (95% CI)	P value	HR (95% CI)	P value
**Univariate Analysis**				
Age	0.994(0.979-1.009)	0.427	0.989(0.973-1.004)	0.152
Sex (male versus female)	0.861(0.574-1.293)	0.470	0.902(0.592-1.373)	0.630
Serum AFP (> 20 versus ≤ 20 ng/ml)	1.418(0.927-2.170)	0.108	1.301(0.849-1.995)	0.227
HBV infection (yes versus no)	2.114(1.427-3.222)	< 0.001	2.111(1.403-3.178)	< 0.001
Cirrhosis (present versus absent)	1.039(0.741-1.456)	0.825	1.132(0.798-1.606)	0.488
Child-pugh score (B versus A)	1.254(0.835-1.884)	0.274	1.246(0.824-1.886)	0.297
Tumor number (multiple versus single)	2.596(1.903-3.540)	**< 0.001**	2.920(2.131-4.000)	**< 0.001**
Maximal tumor size (> 5 versus ≤ 5 cm)	1.472(1.084-1.998)	**0.013**	1.437(1.052-1.963)	**0.023**
Tumor encapsulation (present versus absent)	0.341(0.248-0.469)	**< 0.001**	0.326(0.236-0.450)	**< 0.001**
Microvascular invasion (present versus absent)	2.338(1.720-3.179)	**< 0.001**	2.470(1.806-3.377)	**< 0.001**
Tumor differentiation (III-IV versus I-II)	3.032(2.193-4.191)	**< 0.001**	3.163(2.284-4.380)	**< 0.001**
TNM stage (III versus I-II)	6.289(4.444-8.901)	**< 0.001**	6.923(4.885-9.809)	**< 0.001**
BACH1 (positive versus negative)	2.820(2.052-3.876)	**< 0.001**	2.595(1.879-3.583)	**< 0.001**
**Multivariate analysis1**				
Tumor number (multiple versus single)	1.223(0.802-1.864)	0.349	1.429(0.939-2.176)	0.096
Maximal tumor size (> 5 versus ≤ 5 cm)	0.838(0.592-1.184)	0.316	0.843(0.590-1.204)	0.347
Tumor encapsulation (present versus absent)	0.738(0.489-1.112)	0.146	0.781(0.515-1.184)	0.245
Microvascular invasion (present versus absent)	1.428(0.997-2.047)	0.052	1.581(1.095-2.284)	0.015
Tumor differentiation (III-IV versus I-II)	1.145(0.733-1.791)	0.552	1.124(0.710-1.778)	0.619
TNM stage (III versus I-II)	3.494(2.021-6.043)	**< 0.001**	3.755(2.165-6.512)	**< 0.001**
BACH1 (positive versus negative)	1.940(1.345-2.798)	**< 0.001**	1.660(1.145-2.407)	**0.007**

Supplementary Table S2 Univariate and Multivariate Analysis of Factors Associated with Time to Recurrence and Overall Survival in Cohort II HCC Patients (n = 210)

Clinical Variables	Time To Recurrence		Overall Survival	
	HR (95% CI)	P value	HR (95% CI)	P value
**Univariate Analysis**				
Age	0.987(0.970-1.004)	0.122	0.985(0.968-1.002)	0.076
Sex (male versus female)	0.769(0.499-1.184)	0.232	0.722(0.468-1.115)	0.142
Serum AFP (> 20 versus ≤ 20 ng/ml)	1.199(0.788-1.825)	0.397	1.248(0.809-1.927)	0.316
HBV infection (yes versus no)	0.822(0.537-1.259)	0.367	0.810(0.525-1.250)	0.340
Cirrhosis (present versus absent)	0.873(0.595-1.282)	0.489	0.860(0.583-1.271)	0.450
Child-pugh score (B versus A)	0.973(0.649-1.460)	0.896	0.981(0.649-1.482)	0.927
Tumor number (multiple versus single)	1.984(1.398-2.817)	**< 0.001**	2.068(1.446-2.957)	**< 0.001**
Maximal tumor size (> 5 versus ≤ 5 cm)	1.328(0.934-1.888)	0.114	1.418(0.988-2.035)	0.058
Tumor encapsulation (present versus absent)	0.354(0.249-0.505)	**< 0.001**	0.324(0.226-0.466)	**< 0.001**
Microvascular invasion (present versus absent)	2.319(1.629-3.301)	**< 0.001**	2.572(1.790-3.695)	**< 0.001**
Tumor differentiation (III-IV versus I-II)	2.128(1.422-3.184)	**< 0.001**	2.319(1.545-3.481)	**< 0.001**
TNM stage (III versus I-II)	7.507(4.967-11.345)	**< 0.001**	8.062(5.306-12.250)	**< 0.001**
BACH1 (positive versus negative)	2.527(1.748-3.652)	**< 0.001**	2.416(1.662-3.513)	**< 0.001**
**Multivariate analysis1**				
Tumor number (multiple versus single)	0.929(0.548-1.575)	0.784	1.023(0.594-1.761)	0.935
Maximal tumor size (> 5 versus ≤ 5 cm)	0.910(0.592-1.399)	0.667	0.952(0.607-1.492)	0.830
Tumor encapsulation (present versus absent)	0.560(0.336-0.934)	0.026	0.577(0.340-0.979)	0.041
Microvascular invasion (present versus absent)	1.051(0.623-1.772)	0.853	1.184(0.692-2.026)	0.538
Tumor differentiation (III-IV versus I-II)	1.264(0.807-1.980)	0.306	1.458(0.929-2.289)	0.101
TNM stage (III versus I-II)	5.876(3.211-10.752)	**< 0.001**	5.913(3.179-11.000)	**< 0.001**
BACH1 (positive versus negative)	1.943(1.308-2.887)	**0.001**	1.793(1.203-2.672)	**0.004**

Supplementary Table S3. List of genes differentially expressed in PLC/PRF/5-LV-BACH1 cells versus PLC/PRF/5-LV-Control cells using Human Cell Motility PCR Array

Gene	PLC/PRF/5-LV-BACH1 vs PLC/PRF/5-LV-Control	Description
IGF1R	7.51	Insulin-like growth factor 1 receptor
PTK2	6.63	PTK2 protein tyrosine kinase 2
ACTR2	5.87	ARP2 actin-related protein 2 homolog (yeast)
WASF2	5.53	WAS protein family, member 2
MMP9	4.28	Matrix metallopeptidase 9 (gelatinase B, 92kDa gelatinase, 92kDa type IV collagenase)
STAT3	3.94	Signal transducer and activator of transcription 3 (acute-phase response factor)
ROCK1	3.67	Rho-associated, coiled-coil containing protein kinase 1
ITGB1	3.52	Integrin, beta 1 (fibronectin receptor, beta polypeptide, antigen CD29 includes MDF2, MSK12)
EGFR	3.23	Epidermal growth factor receptor
RAC1	2.84	Ras-related C3 botulinum toxin substrate 1 (rho family, small GTP binding protein Rac1)
MAPK1	2.57	Mitogen-activated protein kinase 1
PAK1	2.31	P21 protein (Cdc42/Rac)-activated kinase 1
MYH9	2.19	Myosin, heavy chain 9, non-muscle
EGF	2.08	Epidermal growth factor
MET	2.03	Met proto-oncogene (hepatocyte growth factor receptor)
CDC42	1.99	Cell division cycle 42 (GTP binding protein, 25kDa)
RASA1	1.97	RAS p21 protein activator (GTPase activating protein) 1
ARF6	1.95	ADP-ribosylation factor 6
ARHGEF7	1.94	Rho guanine nucleotide exchange factor (GEF) 7
WASF1	1.90	WAS protein family, member 1
AKT1	1.89	V-akt murine thymoma viral oncogene homolog 1
EZR	1.88	Ezrin
PXN	1.85	Paxillin
SH3PXD2A	1.82	SH3 and PX domains 2A
ACTN4	1.81	Actinin, alpha 4
BCAR1	1.78	Breast cancer anti-estrogen resistance 1
SVIL	1.73	Supervillin
CSF1	1.71	Colony stimulating factor 1 (macrophage)
PLCG1	1.67	Phospholipase C, gamma 1
PTEN	1.66	Phosphatase and tensin homolog
SRC	1.63	V-src sarcoma (Schmidt-Ruppin A-2) viral oncogene homolog (avian)
WIPF1	1.60	WAS/WASL interacting protein family, member 1
MYH10	1.58	Myosin, heavy chain 10, non-muscle
VASP	1.54	Vasodilator-stimulated phosphoprotein
CAV1	1.51	Caveolin 1, caveolae protein, 22kDa
LIMK1	1.47	LIM domain kinase 1
RAC2	1.47	Ras-related C3 botulinum toxin substrate 2 (rho family, small GTP binding protein Rac2)
PAK4	1.46	P21 protein (Cdc42/Rac)-activated kinase 4
RHOC	1.42	Ras homolog gene family, member C
ARHGDIA	1.39	Rho GDP dissociation inhibitor (GDI) alpha

DPP4	1.34	Dipeptidyl-peptidase 4
RND3	1.34	Rho family GTPase 3
FGF2	1.27	Fibroblast growth factor 2 (basic)
ITGB2	1.25	Integrin, beta 2 (complement component 3 receptor 3 and 4 subunit)
MMP14	1.23	Matrix metallopeptidase 14 (membrane-inserted)
FAP	1.18	Fibroblast activation protein, alpha
HGF	1.15	Hepatocyte growth factor (hepapoietin A; scatter factor)
MMP2	1.11	Matrix metallopeptidase 2 (gelatinase A, 72kDa gelatinase, 72kDa type IV collagenase)
BAIAP2	1.07	BAI1-associated protein 2
IGF1	1.04	Insulin-like growth factor 1 (somatomedin C)
RHO	1.0	Rhodopsin
MYL9	-1.02	Myosin, light chain 9, regulatory
RHOB	-1.04	Ras homolog gene family, member B
ACTN3	-1.07	Actinin, alpha 3
PFN1	-1.10	Profilin 1
TGFB1	-1.14	Transforming growth factor, beta 1
PLAUR	-1.17	Plasminogen activator, urokinase receptor
VIM	-1.18	Vimentin
TIMP2	-1.23	TIMP metallopeptidase inhibitor 2
DIAPH1	-1.25	Diaphanous homolog 1 (Drosophila)
PTK2B	-1.27	PTK2B protein tyrosine kinase 2 beta
ITGB3	-1.32	Integrin, beta 3 (platelet glycoprotein IIIa, antigen CD61)
CTTN	-1.35	Cortactin
VCL	-1.38	Vinculin
ILK	-1.41	Integrin-linked kinase
ACTN1	-1.42	Actinin, alpha 1
MYLK	-1.42	Myosin light chain kinase
CAPN2	-1.47	Calpain 2, (m/II) large subunit
CFL1	-1.56	Cofilin 1 (non-muscle)
PLD1	-1.59	Phospholipase D1, phosphatidylcholine-specific
CAPN1	-1.63	Calpain 1, (mu/I) large subunit
TLN1	-1.67	Talin 1
ITGA4	-1.72	Integrin, alpha 4 (antigen CD49D, alpha 4 subunit of VLA-4 receptor)
CRK	-1.86	V-crk sarcoma virus CT10 oncogene homolog (avian)
MSN	-1.93	Moesin
WASL	-2.03	Wiskott-Aldrich syndrome-like
PRKCA	-2.18	Protein kinase C, alpha
PTPN1	-2.34	Protein tyrosine phosphatase, non-receptor type 1
VEGFA	-2.43	Vascular endothelial growth factor A
ENAH	-2.51	Enabled homolog (Drosophila)
RHOA	-2.78	Ras homolog gene family, member A
RDX	-3.21	Radixin
ACTR3	-3.42	ARP3 actin-related protein 3 homolog (yeast)
PIK3CA	-4.33	Phosphoinositide-3-kinase, catalytic, alpha polypeptide

Supplementary Table S4. List of genes differentially expressed in MHCC97H-shBACH1 cells versus MHCC97H-shControl cells using Human Cell Motility PCR Array

Gene	MHCC97H-shBACH1 vs MHCC97H-shControl	Description
PTK2	-6.88	PTK2 protein tyrosine kinase 2
IGF1R	-5.63	Insulin-like growth factor 1 receptor
CAV1	-4.72	Caveolin 1, caveolae protein, 22kDa
MMP9	-3.56	Matrix metallopeptidase 9 (gelatinase B, 92kDa gelatinase, 92kDa type IV collagenase)
CSF1	-3.45	Colony stimulating factor 1 (macrophage)
MYH9	-3.23	Myosin, heavy chain 9, non-muscle
WASF1	-2.87	WAS protein family, member 1
ROCK1	-2.63	Rho-associated, coiled-coil containing protein kinase 1
DPP4	-2.58	Dipeptidyl-peptidase 4
ARF6	-2.51	ADP-ribosylation factor 6
EGF	-2.47	Epidermal growth factor
ACTR2	-2.43	ARP2 actin-related protein 2 homolog (yeast)
PXN	-2.36	Paxillin
SRC	-2.29	V-src sarcoma (Schmidt-Ruppin A-2) viral oncogene homolog (avian)
PAK1	-2.11	P21 protein (Cdc42/Rac)-activated kinase 1
MMP14	-2.08	Matrix metallopeptidase 14 (membrane-inserted)
CDC42	-2.04	Cell division cycle 42 (GTP binding protein, 25kDa)
ACTN4	-2.01	Actinin, alpha 4
MAPK1	-1.98	Mitogen-activated protein kinase 1
ACTN1	-1.95	Actinin, alpha 1
CTTN	-1.95	Cortactin
ACTR3	-1.87	ARP3 actin-related protein 3 homolog (yeast)
ILK	-1.84	Integrin-linked kinase
MET	-1.81	Met proto-oncogene (hepatocyte growth factor receptor)
PTEN	-1.77	Phosphatase and tensin homolog
RHO	-1.73	Rhodopsin
ITGB1	-1.64	Integrin, beta 1 (fibronectin receptor, beta polypeptide, antigen CD29 includes MDF2, MSK12)
CFL1	-1.62	Cofilin 1 (non-muscle)
RHOB	-1.57	Ras homolog gene family, member B
STAT3	-1.54	Signal transducer and activator of transcription 3 (acute-phase response factor)
MSN	-1.53	Moesin
TIMP2	-1.52	TIMP metallopeptidase inhibitor 2
MYL9	-1.49	Myosin, light chain 9, regulatory
ARHGEF7	-1.46	Rho guanine nucleotide exchange factor (GEF) 7
LIMK1	-1.46	LIM domain kinase 1
RAC1	-1.43	Ras-related C3 botulinum toxin substrate 1 (rho family, small GTP binding protein Rac1)
RND3	-1.40	Rho family GTPase 3
VIM	-1.39	Vimentin
MMP2	-1.37	Matrix metallopeptidase 2 (gelatinase A, 72kDa gelatinase, 72kDa type IV collagenase)
HGF	-1.34	Hepatocyte growth factor (hepapoietin A; scatter factor)

WASF2	-1.33	WAS protein family, member 2
DIAPH1	-1.32	Diaphanous homolog 1 (Drosophila)
BCAR1	-1.30	Breast cancer anti-estrogen resistance 1
PLCG1	-1.29	Phospholipase C, gamma 1
EGFR	-1.27	Epidermal growth factor receptor
PTK2B	-1.27	PTK2B protein tyrosine kinase 2 beta
RHOC	-1.25	Ras homolog gene family, member C
VASP	-1.23	Vasodilator-stimulated phosphoprotein
RASA1	-1.21	RAS p21 protein activator (GTPase activating protein) 1
PIK3CA	-1.19	Phosphoinositide-3-kinase, catalytic, alpha polypeptide
PRKCA	-1.17	Protein kinase C, alpha
CRK	-1.14	V-crk sarcoma virus CT10 oncogene homolog (avian)
TGFB1	-1.08	Transforming growth factor, beta 1
RHOA	-1.06	Ras homolog gene family, member A
TLN1	-1.03	Talin 1
SVIL	1.02	Supervillin
PAK4	1.07	P21 protein (Cdc42/Rac)-activated kinase 4
WIPF1	1.11	WAS/WASL interacting protein family, member 1
FAP	1.18	Fibroblast activation protein, alpha
ACTN3	1.23	Actinin, alpha 3
ITGB3	1.28	Integrin, beta 3 (platelet glycoprotein IIIa, antigen CD61)
PLD1	1.32	Phospholipase D1, phosphatidylcholine-specific
RAC2	1.32	Ras-related C3 botulinum toxin substrate 2 (rho family, small GTP binding protein Rac2)
SH3PXD2A	1.34	SH3 and PX domains 2A
WASL	1.38	Wiskott-Aldrich syndrome-like
AKT1	1.44	V-akt murine thymoma viral oncogene homolog 1
CAPN2	1.49	Calpain 2, (m/II) large subunit
ENAH	1.51	Enabled homolog (Drosophila)
ITGB2	1.57	Integrin, beta 2 (complement component 3 receptor 3 and 4 subunit)
MYLK	1.62	Myosin light chain kinase
PLAUR	1.78	Plasminogen activator, urokinase receptor
ARHGDIA	1.79	Rho GDP dissociation inhibitor (GDI) alpha
BAIAP2	1.82	BAI1-associated protein 2
MYH10	1.88	Myosin, heavy chain 10, non-muscle
PTPN1	1.94	Protein tyrosine phosphatase, non-receptor type 1
VEGFA	1.99	Vascular endothelial growth factor A
RDX	2.21	Radixin
CAPN1	2.38	Calpain 1, (mu/I) large subunit
ITGA4	2.43	Integrin, alpha 4 (antigen CD49D, alpha 4 subunit of VLA-4 receptor)
PFN1	2.57	Profilin 1
IGF1	3.31	Insulin-like growth factor 1 (somatomedin C)
VCL	3.32	Vinculin
FGF2	3.86	Fibroblast growth factor 2 (basic)
EZR	4.07	Ezrin

Supplementary Table S5 Correlation between IGF1R expression and clinicopathological characteristics of HCCs in two independent cohorts of human HCC tissues

Clinicopathological variables		Cohort I: Tumor IGF1R expression Negative (n = 128)	Cohort I: Tumor IGF1R expression Positive (n = 152)	Cohort I: *P* Value	Cohort II: Tumor IGF1R expression Negative (n = 102)	Cohort II: Tumor IGF1R expression Positive (n = 108)	Cohort II: *P* Value
Age		52.04 （9.342）	52.32 （10.586）	0.818	52.92 （9.974）	52.50 （11.130）	0.773
Sex	female	21	24	1.000	20	19	0.726
	male	107	128		82	89	
Serum AFP	≤ 20ng/ml	22	27	1.000	26	24	0.628
	> 20ng/ml	106	125		76	84	
Virus infection	HBV	81	112	0.172	79	79	0.280
	HCV	26	18		7	12	
	HBV + HCV	9	7		7	3	
	none	12	15		9	14	
Cirrrhosis	absent	37	42	0.894	26	31	0.643
	present	91	110		76	77	
Child-pugh score	Class A	106	130	0.622	75	83	0.633
	Class B	22	22		27	25	
Tumor number	single	104	87	**< 0.001**	73	51	**< 0.001**
	multiple	24	65		29	57	
Maximal tumor size	≤ 5cm	89	73	**< 0.001**	47	52	0.784
	> 5cm	39	79		55	56	
Tumor encapsulation	absent	13	62	**< 0.001**	26	60	**< 0.001**
	present	115	90		76	48	
Microvascular invasion	absent	103	69	**< 0.001**	75	41	**< 0.001**
	present	25	83		27	67	
Tumor differentiation	I-II	121	86	**< 0.001**	95	72	**< 0.001**
	III-Ⅳ	7	66		7	36	
TNM stage	I-II	122	101	**< 0.001**	97	73	**< 0.001**
	III	6	51		5	35	

Supplementary Table S6 Correlation between PTK2 expression and clinicopathological characteristics of HCCs in two independent cohorts of human HCC tissues

		Cohort I			Cohort II		
		Tumor PTK2 expression			Tumor PTK2 expression		
Clinicopathological variables		Negative (n = 142)	Positive (n = 138)	*P* Value	Negative (n = 100)	Positive (n = 110)	*P* Value
Age		52.77（9.432）	51.59（10.591）	0.322	52.81（10.124）	52.61（10.989）	0.891
Sex	female	23	22	1.000	19	20	1.000
	male	119	116		81	90	
Serum AFP	≤ 20ng/ml	27	22	0.532	29	21	0.106
	> 20ng/ml	115	116		71	89	
Virus infection	HBV	89	104	0.102	80	78	0.192
	HCV	28	16		10	9	
	HBV + HCV	8	8		3	7	
	none	17	10		7	16	
Cirrrhosis	absent	40	39	1.000	26	31	0.758
	present	102	99		74	79	
Child-pugh score	Class A	121	115	0.743	81	77	0.079
	Class B	21	23		19	33	
Tumor number	single	121	70	**< 0.001**	69	55	**0.007**
	multiple	21	68		31	55	
Maximal tumor size	≤ 5cm	89	73	0.116	52	47	0.213
	> 5cm	53	65		48	63	
Tumor encapsulation	absent	14	61	**< 0.001**	27	59	**< 0.001**
	present	128	77		73	51	
Microvascular invasion	absent	114	58	**< 0.001**	71	45	**< 0.001**
	present	28	80		29	65	
Tumor differentiation	I-II	135	72	**< 0.001**	92	75	**< 0.001**
	III-Ⅳ	7	66		8	35	
TNM stage	I-II	137	86	**< 0.001**	89	81	**0.005**
	III	5	52		11	29	

Supplementary Table S7. Primer sequences used in the study

Primer name	Primer sequences	Enzyme
**Primers for real-time PCR:**		
BACH1 sense:	5'-TCTGAGTGAGAACTCGGTTTTTG-3'	
BACH1 antisense:	5'-CGCTGGTCATTAAGGCTGAGTAA-3'	
IGF1R sense:	5’-AGGATATTGGGCTTTACAACCTG-3’	
IGF1R antisense:	5’-GAGGTAACAGAGGTCAGCATTTT-3’	
PTK2 sense:	5’-TGGTGCAATGGAGCGAGTATT-3’	
PTK2 antisense:	5’-CAGTGAACCTCCTCTGACCG-3’	
β-actin sense:	5’-CATGTACGTTGCTATCCAGGC-3’	
β-actin antisense:	5’-CTCCTTAATGTCACGCACGAT-3’	
**Primers for IGF1R promoter construct:**		
(-2128/+70) IGF1R sense:	5’-TATAGGTACCCTCCAGAGTGGATCTGCA-3’	KpnI
(-1720/+70) IGF1R sense:	5’-TATAGGTACCACGATGGATACACGTTCT-3’	KpnI
(-572/+70) IGF1R sense:	5’-TATAGGTACCTTTCCAGTACGCAGCGAA-3’	KpnI
Antisense:	5’-ATATACGCGTCTCAGCGGAGTTAATGCT-3’	MluI
**Primers for IGF1R promoter site-directed mutagenesis:**		
BAHC1 binding site:		
binding site 2 mutation sense:	5’-TTTCTATACAACT**acag**CTGAATTGAGCTA-3’	
binding site 2 mutation antisense:	5’-TAGCTCAATTCAG**ctgt**AGTTGTATAGAAA-3’	
binding site 1 mutation sense:	5’-GCATCAATTAGCC**caga**TCATGAAACCGGA-3’	
binding site 1 mutation antisense:	5’-TCCGGTTTCATGA**tctg**GGCTAATTGATGC-3’	
**Primers used for ChIP in the IGF1R promoter:**		
distant region sense:	5’-GTTTCTGCTCCAAAAGAG-3’	
distant region antisense:	5’-AAAGGCTAGTGCTAATAT-3’	
binding site 1 sense:	5’-CCCGATTGTTTGAGACAA-3’	
binding site 1 antisense:	5’-TAGGACTGCCATCTAATG-3’	
**Primers for PTK2 promoter construct:**		
(-2607/+155) PTK2 sense:	5’-TATAGGTACCCTGGGATTACAGGCACGT-3’	KpnI
(-2204/+155) PTK2 sense:	5’-TATAGGTACCATGCTAAGCACCCTGCTG-3’	KpnI
(-1797/+155) PTK2 sense:	5’-TATAGGTACCGGCTCAGAGACATTATAC-3’	KpnI
(-575/+155) PTK2 sense:	5’-TATAGGTACCCATGGAGAGGCAATTCCT-3’	KpnI
Antisense:	5’-ATATACGCGTGGACTTAGAAGTCCACTG-3’	MluI
**Primers for PTK2 promoter site-directed mutagenesis:**		
BACH1 binding site:		
binding site 3 mutation sense:	5’-GTCAGCCTTCCTT**acag**CTGTGCTCTGGTG-3’	
binding site 3 mutation antisense:	5’-CACCAGAGCACAG**ctgt**AAGGAAGGCTGAC-3’	
binding site 2 mutation sense:	5’-CCGTTCTCCTGCC**gaca**CCTCCCGAATAGC-3’	
binding site 2 mutation antisense:	5’-GCTATTCGGGAGG**tgtc**GGCAGGAGAACGG-3’	
binding site 1 mutation sense:	5’-TTACTCCTAAACC**caga**TTCTCAGGCACCA-3’	
binding site 1 mutation antisense:	5’-TGGTGCCTGAGAA**tctg**GGTTTAGGAGTAA-3’	
**Primers used for ChIP in the PTK2 promoter:**		
distant region sense:	5’-CCATCTGGTGCAGTGCAG-3’	
distant region antisense:	5’-ATTGCTCGAACCCAGGAG-3’	
binding site 1 sense:	5’-CTCCATTTCACAGATGAG-3’	
binding site 1 antisense:	5’-CCTAGGAAACAGTGGCTT-3’	
**Primers for BACH1 promoter construct:**		
(-1996/+237) BACH1 sense:	5’-TATAACGCGTCCAGGTTCAAGCGATTCC-3’	MluI
(-1623/+237) BCAH1 sense:	5’-TATAACGCGTTCAGGCTAGAAAATGTCA-3’	MluI

(-1110/+237) BACH1 sense:	5’-TATAACGCGTATGCAGTCATGTTAGTCG-3’	MluI
(-538/+237) BACH1 sense:	5’-TATAACGCGTAGGAGCTCTTCAAGGGGT-3’	MluI
(-48/+237) BACH1 sense:	5’-TATAACGCGTTGAGTCACCTGACCGCTG-3’	MluI
antisense:	5’-ATATGCTAGCCGCGGCCACTTCCAGGAT-3’	NheI
**Primers for BACH1 promoter site-directed mutagenesis:**		
AP-1 binding site mutation sense:	5’-GTGGTCCTCGAGT**aaag**GTCAGTTCAGGAT-3’	
AP-1 binding site mutation antisense:	5’-ATCCTGAACTGAC**cttt**ACTCGAGGACCAC-3’	
ETS1 binding site mutation sense:	5’-CCCCCGCGTGGGC**ccaa**GGCCGCGGCGACC-3’	
ETS1 binding site mutation antisense:	5’-GGTCGCCGCGGCC**ttgg**GCCCACGCGGGGG-3’	
SP-1 binding site mutation sense:	5’-CTTCAGCGGGCGG**ataa**GGTTTTGGCGCCG-3’	
SP-1 binding site mutation antisense:	5’-CGGCGCCAAAACC**ttat**CCGCCCGCTGAAG-3’	
**Primers used for ChIP in the BACH1 promoter:**		
distant region sense:	5’-GTGCCACATCTTTCACTG-3’	
distant region antisense:	5’-AATGCAGATTCCTGGGTC-3’	
binding site 1 sense:	5’-TTCCGCCCTCAGAAGGGA-3’	
binding site 1 antisense:	5’-GCCTCTGTCAGCGAACGA-3’	
**Primers used for ChIP in the CXCR4 promoter:**		
sense:	5’-GATTCTGCCACTACCAGG-3’	
antisense:	5’-CCAGAGGCATTTCCTAAG-3’	

Supplementary Table S8. Knockdown shRNA sequences used in this study

Gene	Sequence
BACH1	
shRNA-1	GCCCATATGCTTGTGTCATTA
shRNA-2	CCAGCAAGAATGCCCAAGAAA
shRNA-3	CCTATGAATCTTCTGTGCATA
IGF1R	
shRNA-1	GCCGAAGATTTCACAGTCAAA
shRNA-2	GCGGTGTCCAATAACTACATT
shRNA-3	GCCTTTCACATTGTACCGCAT
PTK2	
shRNA-1	GATGTTGGTTTAAAGCGATTT
shRNA-2	CCGATTGGAAACCAACATATA
shRNA-3	CAACAGGTGAAGAGCGATTAT
ETS1	
ShRNA-1	GCCCTGGGTAAAGACTGCTTT
ShRNA-2	CTGGAATTACTCACTGATAAA
ShRNA-3	CCGGATATGGAATGTGCAGAT

Supplementary Table S9 IHC scores of BACH1 in Cohort I HCC Patients (n = 280)

**Patient Number**	**Adjacent Nontumorous Tissues**	**HCC Tissues**
1	2	12
2	3	8
3	1	0
4	2	2
5	8	9
6	3	12
7	2	1
8	1	12
9	8	1
10	6	2
11	2	8
12	0	0
13	0	3
14	0	1
15	6	9
16	4	3
17	12	2
18	8	0
19	2	8
20	2	0
21	1	4
22	4	4
23	3	12
24	2	3
25	9	3
26	6	9
27	8	8
28	12	0
29	2	1
30	0	12
31	0	9
32	0	0
33	1	9
34	1	8
35	2	0
36	4	1
37	2	8
38	3	6
39	8	3
40	1	2
41	1	1
42	2	12
43	3	9
44	6	3
45	2	2
46	6	1
47	1	6

48	8	2
49	12	1
50	9	4
51	0	0
52	2	8
53	3	6
54	8	3
55	1	12
56	1	9
57	12	1
58	6	2
59	8	0
60	3	8
61	2	3
62	2	4
63	1	6
64	1	2
65	1	12
66	6	9
67	4	8
68	4	0
69	8	6
70	9	1
71	6	3
72	3	9
73	3	8
74	2	2
75	8	1
76	12	4
77	6	1
78	4	9
79	2	0
80	2	6
81	4	3
82	1	12
83	8	1
84	3	9
85	9	9
86	4	0
87	0	8
88	1	6
89	2	1
90	1	8
91	3	1
92	1	2
93	0	3
94	0	9
95	0	0
96	3	12

97	8	0
98	3	6
99	2	1
100	0	6
101	0	3
102	1	8
103	0	3
104	9	1
105	12	6
106	4	1
107	0	2
108	0	3
109	1	12
110	3	1
111	1	2
112	9	12
113	3	9
114	2	0
115	0	12
116	2	9
117	4	8
118	8	2
119	2	6
120	1	3
121	3	2
122	1	1
123	1	4
124	8	2
125	3	1
126	4	8
127	6	1
128	2	0
129	1	6
130	0	1
131	0	8
132	3	2
133	3	0
134	1	6
135	1	3
136	12	4
137	9	0
138	1	6
139	1	8
140	2	9
141	2	1
142	9	2
143	4	6
144	6	8
145	2	0

146	1	1
147	0	6
148	2	8
149	3	1
150	8	9
151	1	2
152	2	8
153	1	0
154	6	9
155	8	3
156	9	1
157	6	8
158	0	6
159	9	2
160	4	1
161	3	9
162	8	2
163	4	1
164	3	8
165	3	1
166	2	0
167	8	4
168	1	2
169	3	0
170	9	8
171	1	6
172	8	1
173	12	8
174	0	0
175	0	6
176	1	0
177	8	12
178	9	0
179	4	3
180	3	9
181	3	3
182	2	2
183	1	1
184	6	9
185	4	6
186	6	1
187	0	2
188	0	0
189	1	6
190	2	4
191	3	2
192	4	8
193	2	9
194	3	0

195	8	1
196	2	4
197	9	6
198	1	3
199	2	6
200	0	8
201	0	3
202	3	1
203	4	6
204	8	9
205	2	2
206	2	0
207	1	6
208	9	3
209	12	2
210	1	1
211	9	8
212	1	3
213	0	12
214	0	2
215	1	6
216	2	2
217	6	1
218	4	12
219	8	0
220	3	9
221	1	2
222	8	4
223	2	6
224	1	9
225	9	2
226	1	6
227	2	2
228	3	6
229	4	9
230	2	0
231	1	9
232	0	8
233	3	1
234	3	6
235	2	0
236	8	8
237	3	1
238	9	2
239	0	4
240	12	1
241	8	4
242	8	9
243	9	2

244	4	9
245	6	12
246	6	1
247	8	2
248	3	12
249	1	9
250	2	8
251	2	6
252	3	2
253	0	1
254	8	3
255	1	0
256	2	6
257	6	2
258	4	1
259	3	2
260	2	6
261	4	2
262	0	0
263	0	12
264	1	9
265	1	8
266	1	3
267	2	9
268	4	6
269	1	3
270	2	1
271	6	2
272	12	9
273	0	8
274	8	6
275	2	0
276	1	12
277	3	2
278	9	9
279	6	12
280	3	8

Supplementary Table S10 IHC scores of BACH1 in Cohort II HCC Patients (n = 210)

Patient Number	Adjacent Nontumorous Tissues	HCC Tissues
1	0	2
2	0	4
3	0	9
4	4	0
5	8	2
6	2	2
7	4	12
8	2	3
9	4	9
10	8	2
11	1	1
12	1	12
13	3	1
14	3	2
15	2	9
16	3	1
17	1	9
18	1	12
19	8	3
20	3	6
21	6	0
22	1	8
23	3	2
24	4	2
25	6	3
26	2	1
27	3	6
28	3	2
29	4	8
30	0	9
31	8	3
32	9	8
33	12	9
34	3	2
35	2	8
36	6	3
37	6	3
38	4	9
39	3	12
40	0	4
41	2	1
42	4	9
43	3	9
44	1	2
45	4	0
46	3	8
47	9	2

48	8	1
49	4	3
50	3	9
51	1	2
52	2	12
53	6	2
54	4	8
55	3	3
56	8	2
57	3	3
58	4	9
59	4	12
60	2	1
61	9	0
62	6	2
63	4	2
64	3	9
65	8	3
66	3	8
67	0	3
68	3	12
69	2	3
70	0	8
71	0	2
72	4	0
73	3	9
74	2	9
75	3	2
76	4	3
77	3	8
78	3	2
79	2	1
80	6	12
81	4	9
82	3	2
83	0	0
84	1	8
85	9	2
86	1	3
87	2	9
88	4	4
89	6	2
90	4	3
91	3	8
92	0	6
93	0	1
94	1	8
95	2	9
96	0	1

97	2	9
98	0	0
99	4	8
100	9	0
101	6	9
102	8	3
103	2	2
104	4	9
105	3	8
106	9	1
107	4	9
108	1	2
109	3	2
110	4	6
111	4	2
112	6	3
113	2	6
114	3	9
115	1	2
116	1	9
117	0	6
118	6	1
119	3	12
120	0	8
121	1	0
122	2	0
123	9	8
124	6	12
125	9	2
126	6	3
127	3	8
128	1	3
129	2	9
130	1	0
131	9	0
132	1	9
133	2	8
134	3	2
135	2	9
136	4	2
137	6	3
138	3	1
139	4	8
140	3	9
141	8	3
142	3	3
143	6	0
144	1	4
145	0	2

146	4	3
147	6	8
148	1	3
149	6	9
150	4	0
151	2	0
152	2	3
153	3	8
154	3	0
155	3	12
156	0	9
157	9	3
158	12	8
159	1	0
160	0	9
161	0	8
162	1	6
163	4	2
164	6	3
165	2	3
166	12	6
167	4	3
168	3	6
169	2	4
170	2	4
171	4	3
172	4	6
173	3	2
174	8	1
175	9	2
176	1	12
177	0	9
178	2	8
179	2	2
180	0	8
181	6	0
182	6	3
183	3	8
184	4	12
185	6	4
186	2	8
187	3	1
188	4	9
189	1	9
190	12	8
191	3	1
192	0	1
193	2	6
194	3	3

195	3	9
196	4	3
197	3	12
198	1	8
199	4	1
200	6	9
201	4	3
202	2	0
203	2	8
204	4	0
205	1	3
206	0	4
207	9	1
208	0	6
209	2	0
210	4	6
